# Supplementary material for: Ancient DNA and deep population structure in sub-Saharan African foragers
Source: Nature. 2022 Feb 23;603(7900):290–6. doi: 10.1038/s41586-022-04430-9 (PMC8907066; doi:10.1038/s41586-022-04430-9)
Supplement: Supplementary file 1 — Additional information on sampling and permissions, archaeological context and data analysis. [file 41586_2022_4430_MOESM1_ESM.pdf]

nature portfolio

https://doi.org/10.1038/s41586-022-04430-9

Supplementary information

Ancient DNA and deep population structure in sub-Saharan African foragers

In the format provided by the authors and unedited

SUPPLEMENTARY INFORMATION

Ancient DNA and deep population structure in sub-Saharan African foragers

Mark Lipson, Elizabeth A. Sawchuk, Jessica C. Thompson, Jonas Oppenheimer, Christian A. Tryon, Kathryn L. Ranhorn, Kathryn M. de Luna, Kendra A. Sirak, Iñigo Olalde, Stanley H. Ambrose, John W. Arthur, Kathryn J.W. Arthur, George Ayodo, Alex Bertacchi, Jessica I. Cerezo-Román, Brendan J. Culleton, Matthew C. Curtis, Jacob Davis, Agness O. Gidna, Annalys Hanson, Potiphar Kaliba, Maggie Katongo, Amandus Kwekason, Myra F. Laird, Jason Lewis, Audax Z.P. Mabulla, Fredrick Mapemba, Alan Morris, George Mudenda, Raphael Mwafulirwa, Daudi Mwangomba, Emmanuel Ndiema, Christine Ogola, Flora Schilt, Pamela Willoughby, David K. Wright, Andrew Zipkin, Ron Pinhasi, Douglas J. Kennett, Fredrick Kyalo Manthi, Nadin Rohland, Nick Patterson, David Reich, Mary E. Prendergast

Table of Contents

Supplementary Note 1. ……………………………………………………………………3
Terminology chosen in this study and use of present-day DNA data

Supplementary Note 2. …………………………………………………………………....5
Integrating archaeological, genetic, and linguistic data

Supplementary Note 3. ……………………………………………………………………8
Details of sampled archaeological skeletons

Figure S1. Relationship between Kahora 1 and Kahora 2 infant burials and associated 14C ages in plan view……………………………………………………………………11

Figure S2. Photomicrographs from the Kahora 1 and Kahora 2 burial contexts………..12

Figure S3. Kahora 1 and Kahora 2 burials in stratigraphic context……………………..13

Table S13. Published dates from Kalemba Rockshelter…………………………….......14

Table S14. Human remains and samples collected from Kalemba 15

Table S15. Radiocarbon dates from Kisese II...17

Table S16. Human remains from Kisese II Rockshelter and samples collected..............19

Table S17. Human remains from Mlambalasi Rockshelter and samples collected..........21

Supplementary Note 4. …………………………………………………………………...22
Radiocarbon dating procedures

Supplementary Note 5: …………………………………………………………………...24
Phenotype-associated SNPs and data authenticity

Supplementary Note 6. ……………………………………………………………………26
Admixture graph fitting

Supplementary Note 7. ……………………………………………………………………33
qpfstats

Supplementary References. …………………………………………….……………...35

Supplementary Note 1. Terminology chosen in this study and use of present-day DNA data

In this study, we use the term "ancestry" in accordance with the definition "ancestors are the individuals from whom you are biologically descended, and ancestry is information about them and their genetic relationship to you"[1]. Given that the genealogical connections between individuals (aside from familial relatives) typically reach many generations into the past, we incorporated both ancient and present-day data, with groups selected for each analysis based on the desired comparisons as well as availability and methodological constraints.

We avoid referring to ancient individuals by terms that imply national identity (e.g., Kenyan, Tanzanian, Malawian, Zambian, South African individuals). We may refer to individuals as having been recovered from a particular present-day country or region, but prefer geographic terms (e.g., southern Africa) to geopolitical ones (e.g., South Africa).

We use the term "forager" to describe both living and ancient people who used hunting, fishing, and gathering (collectively: foraging) as their primary mode of subsistence. While we recognise fluidity in subsistence strategies and the problems inherent in defining identity based on subsistence, for the regions of eastern and south-central Africa and the timeframe of focus here (>5 ka), there is no evidence of food production (pastoralism or farming). For later timeframes, we may describe genetic sequences as "associated with" people known from present-day, historical, or archaeological observations and records to have practiced foraging, agriculture, pastoralism, or agro-pastoralism (or simply food production). This does not imply that there is a necessary connection between manner of subsistence and genetic ancestry, but rather offers a conceptual shorthand for referring to the broad-scale populations that contributed genetic material to the individuals we analysed here.

In southern, central, and eastern Africa today, groups who either forage or whose recent ancestors have done so have been shown to have genetic sequences distinctive from their agricultural, pastoral, or agro-pastoral neighbours. However, groups that share strong genetic similarities can also express significant cultural and linguistic diversity. Using ancient DNA, genetic relatedness can be broadly established between living and ancient groups without implying specific cultural continuity.

In this paper, published genetic data from present-day African groups, including Mbuti in central Africa, Ju|'hoansi in southern Africa, and Dinka in northeastern Africa (among others), are used as points of comparison to aid the interpretation of ancient DNA data. We also draw on published work in our choices of comparative groups and interpretation of results: for example, although the Agaw of Ethiopia are not today pastoralists nor do they identify with a history of pastoralism, Agaw-related ancestry is used as a proxy for ancient pastoralist-related ancestry in eastern Africa, based on findings reported by Prendergast et al.[2] Present-day groups were used (in some cases) as comparators because their ancestry includes genetic sequences from lineages that also had some of these sequences in the past. Different analyses used present-day and/or ancient data depending on the availability of reference groups, genetic similarity to lineages of interest, and/or methodological considerations. However, by including present-day groups in this paper, we do not imply that they represent relict populations, nor that their ancestors have been geographically, socially, or culturally static in the intervening thousands of years.

Following San council recommendations, we use the population-specific term Ju|’hoansi when describing data from this specific group, but we use the term San when speaking more generally about present-day groups with ancestry specific to southern African foragers. For detailed discussion of issues surrounding terminology, see Schlebusch.[3] Finally, we note that when discussing southern African foragers in the manuscript, we are often considering genetic data from both ancient and present-day foragers; where only ancient individuals are being considered, we designate this with the term AncSA (ancient southern Africa).

Supplementary Note 2. Integrating archaeological, genetic, and linguistic data

Archaeological, skeletal, linguistic, historical, and genetic data all indicate massive demographic changes across sub-Saharan Africa within the last 5,000 years (5 ka). These are associated with the invention or introduction and spread of novel technologies (e.g., ceramics, metalworking) and foodways (pastoralism and agriculture), population expansions, the rise of urbanism and states, and displacement and genocide resulting from enslavement and imperialism. Genetic data from contemporary African groups who are presently or historically associated with foraging (hunting, gathering, and/or fishing) lifeways have revealed diversity and some of the most distinctive and deeply divergent human lineages[4–15]. However, more recent demographic transformations have made it impossible to reconstruct ancient population structure and history during the earlier part of the Holocene (the period <12 ka) and Late Pleistocene (the period ~125 – 12 ka) solely based on patterns of genetic variation in present-day Africans living in the same regions[16]. Consequently, studying population history beyond ~2 ka is greatly helped by analysing ancient DNA (aDNA).

The few available aDNA sequences from Holocene sub-Saharan African contexts associated with foraging have already proven valuable in enabling modelling of population dynamics into the Middle Pleistocene[17–19], but they also raise new questions about the timing of gene flow and the distances over which it occurred. When aDNA data are combined with archaeological and linguistic information on patterns of change in subsistence, land use, cultural expression, and social interactions, it becomes feasible to develop hypotheses for, and test hypotheses from, the archaeological and historical linguistic records. We infer that all individuals in this study practiced foraging lifeways based on a combination of their archaeological context and antiquity (pre-dating the spread of food production in these regions). They are all associated with material culture traditionally described by archaeologists as some manifestation of the Later Stone Age (LSA).

While diachronic change and local variation in the patterning of human interactions is evident in the LSA archaeological record, little is known about social networks across Pleistocene Africa. Geochemical sourcing indicates movement of obsidian for stone tools by ~300 ka, culminating in the development of elaborate exchange networks focused on culturally preferred sources of raw materials by the Holocene[20–26]. The lack of obsidian outside the Eastern African Rift System presents challenges for reconstructing exchange elsewhere, however, strontium analysis of ostrich eggshell (OES) beads in southern Africa indicate they were travelling >325km by ~33 ka[27]. Although the intensification of long-distance exchange networks is a signature of the LSA archaeological record[20,28], the extent to which people were moving with objects and how behaviour varied across space and time remain unclear. Our genetic results are consistent with a scenario in which human population movements and interactions aided in the establishment and expansion of LSA exchange networks, forming a population structure that persisted well after behavioural patterns changed as reflected in the reduction in gene flow distances by at least ~16 ka.

Increasing evidence for symbolic expression at forager sites over the Late Pleistocene and Holocene (implicating more complex social networks with more social partners), and the appearance and disappearance of specific stone, bone, and shell artefact types, have been

interpreted as reflecting changes in population densities[21,29–32]. However, palaeodemography often relies on archaeological proxies that are affected by taphonomic bias and bear a complex relationship to numbers of people[33]. Other sources of evidence for or predications about Late Pleistocene population increases and movements include palaeoclimate maps[34] and present-day DNA data[5,12,35]. Our genetic estimates of recent effective population size, although limited by the available data, range from <500 to >2000. While this is consistent with group sizes for at least some present-day African foragers[36], the demographic transformations of the past few centuries have impacted foragers as such that they may not be good points of comparison (e.g., [37]).

Using a larger aDNA dataset supported by multiple Late Pleistocene and early Holocene dates, we demonstrated that central African-related ancestry (closest to present-day Mbuti among sampled populations), along with Mota-related and southern Africa-related ancestry, was widespread in groups ranging from SW Kenya to SE Zambia, with all three components present by ~7 ka in Tanzania and ~16 ka in Malawi. This suggests substantial prior interaction and admixture among populations ancestral to the individuals analysed here, resulting in a genetic signature that was once widespread from the eastern coast to the interior across eastern and south-central Africa. Within this broad three-way population structure, we observe distinct regional genetic clusters. Individuals from Kenya and Tanzania who fall into the same cluster show excess allele sharing beyond what would be expected from having similar ancestry proportions, which could only be explained by post-admixture gene flow and genetic drift within each sub-region. By contrast, for individuals from Malawi and Zambia, the only elevated relatedness is between individuals buried at the same site. This could suggest co-residence of closely related individuals, differing substantially from practices among most presently and historically foraging groups[38].

While there is evidence for ongoing regional gene flow within Kenya and Tanzania during the Holocene, the signature from northern Malawi and Zambia is intensely local. In northern Malawi at Fingira this relatedness spans at least ~3600 years, while at Hora 1 (100 km to the south), it spans at least ~1000 years but not (that we can detect) the ~5000-7000 years separating the earlier and later sampled individuals. This offers evidence for both very long periods of habitation of a region by a population, as well as episodes of population turnover. Other individuals buried at similar times but at sites in southern Malawi and Zambia are not more closely related to each other than they are to the individuals from Kenya and Tanzania, implying regional diversity in the degree of regionalised behaviour. We also observe distinct population histories among Hadza, Sandawe, and ancient eastern African groups, demonstrating intra-regional complexity through time and reiterating the need for caution in ethnographic analogies[39].

Ancient genetic data add weight to bioarchaeological, archaeological, and linguistic arguments for changing spheres of interaction especially across the Late Pleistocene-Holocene boundary. Variability in patterns of gene flow in different regions, and within single regions through time, demonstrates the heterogeneity of ancient forager social landscapes. While based on few individuals, bioarchaeological studies also emphasise deep population structure across the continent during the Late Pleistocene[40–43]. At the regional level, models based on skeletal morphology in southern and south-central Africa suggest increasingly isolated local populations from the Last Glacial Maximum (LGM; ~20 ka) until late Holocene expansions associated with

food production[44,45]. Studies of skeletal morphology at sites in Malawi, including those included here, indicate discontinuities between Holocene groups in eastern and south-central Africa versus southern Africa[44]. Such evidence resonates with concomitant processes of 'regionalisation', a term sometimes used to describe the appearance of distinctive material culture traditions such as the Robberg, Eburran, Nachikufan, and others during the LSA[21,46,47]. While regional technological variation does not necessarily entail genetic variation, diverse lines of evidence imply changes in interaction dynamics in this period. Faunal data indicate subsistence intensification after the LGM[25,48], potentially reflecting responses to climate change, population increases, and/or reduced mobility.

Finally, linguistic data indicate shifts toward more localized interactions. Today, communities presently or historically associated with foraging in central, eastern, and southern Africa speak languages of different families (adopted from relatively recent arrivals in the case of central Africa) indicating a shift away from long-distance interactions in favour of local connections, at least during the Holocene. However, in southern Africa, it is now clear that regional cross-linguistic interaction and borrowing was of such intensity that earlier linguists mistakenly grouped so-called 'Khoe' languages in a single family[49,50]. Intriguingly, one proposed southern African language grouping, Khoe-Kwadi-Sandawe, might indicate longer-distance patterns of migration or interaction between eastern and southern Africa, though there is no way to date such connections from the linguistic data[49,50]. Going forward, detailed local palaeoenvironmental and archaeological research will be essential for developing and testing hypotheses about drivers of regionally distinct dynamics developed from all these lines of data; for example by using behavioural ecological models to predict how the distribution and density of resources structured forager interactions, territoriality, and social boundaries[51–53].

Supplementary Note 3. Details of sampled archaeological skeletons

Permissions

The two Hora 1 burials were recovered in 2019 and are curated by the Malawi Department of Museums and Monuments (formerly Department of Antiquities), now under the Ministry of Youth, Sports, and Culture. Additional sampled individuals from Mazinga and Hora were recovered in 2017 and 2018. The individuals from Fingira and Mtuzi/Chencherere II were recovered during 2016 fieldwork or retrieved from the National Repository in Nguludi, respectively. Permission for the research, including both excavation and ancient DNA protocols for both failed and successful samples, was provided under permits A/III/3.3/70, A/III/3.3/71, AD/23/56, and NCST/RTT/2/6. Export was provided under A/1/1/1/3.6/50, A/1/1/1/3.6/44, A/II/1.5/33, and MHQ/CUL/1/04/2.

The Kalemba Rockshelter burials are curated at the Livingstone Museum, Zambia. Permissions to sample and export skeletal remains for destructive sampling were received from the Director of the Livingstone Museum and from the National Heritage Conservation Commission (Permit NHCC/8WR/004/17).

The Kisese II Rockshelter burials are currently curated in the National Museums of Kenya in Nairobi. Permission to sample these remains was obtained in both Kenya and Tanzania: in Kenya from the National Commission for Science, Technology, and Innovation (NACOSTI permit P/17/34239/17088 issued to MEP), and through affiliation with the National Museums of Kenya (NMK); and in Tanzania the Commission for Science and Technology (COSTECH permits 2017-220/221/222-NA-2012-50 issued to MEP/EAS/JO). Permission to export the skeletal samples for destructive sampling was issued by the Cabinet Secretary, Ministry of Sports and Heritage, Kenya.

The Mlambalasi Rockshelter burials are curated at the National Museum and House of Culture in Dar es Salaam, Tanzania. Permission to sample these remains was granted by the Commission for Science and Technology (COSTECH permits 2017-220/221/222-NA-2012-50 issued to MEP/EAS/JO), through affiliation with the National Museums of Tanzania (NMT). Permission to export the skeletal samples for destructive sampling was granted by the Division of Antiquities, Ministry of Natural Resources and Tourism (Export License 03/2018/2019).

Skeletal sampling protocols

The phalanx from Fingira was recovered while sorting the 3mm fraction of faunal remains excavated in a test excavation in 2016. The site is known to contain commingled human remains from individuals of different developmental ages, so it was impossible to assign it to an individual without genetic sampling. It was photographed before sampling, and as it was from a small juvenile, it was processed in its entirety. The two Hora individuals were named Kahora 1 and Kahora 2 (“little Hora”, as described by our Ngoni excavation team members, to differentiate them from the adult burials recovered from the site). A basic skeletal inventory was conducted in the field, and only a single petrous bone was exported from each. We selected the petrous with the most fragmentary morphology, to preserve the antimere with the more complete morphology (right petrous from Kahora 1, MALAPP plotted find #47625, I19528 in this paper, and right petrous from Kahora 2, MALAPP plotted find #77448, I19529 in this paper). Both

were photographed and scanned using a Nikon XT H 225 ST µCT system with Inspect-X software prior to any destructive analysis. All failed samples from Hora 1, Mazinga 1, and Fingira were from isolated skeletal elements recovered as sieved or plotted finds during excavation, with the exception of the individual labelled "Hora 3", which represents the fragmentary remains of a single adult individual. Each specimen was photographed prior to destructive sampling.

Samples from Kalemba, Kisese II, and Mlambalasi Rockshelters were selected following protocols outlined in detail by Prendergast & Sawchuk[54]. In brief:

1) Sites and skeletons were chosen based upon several factors including their relevance to the research questions, skeletal preservation, and available contextual information.
2) Two skeletal tissue samples (bone and/or tooth) were selected per individual. The second sample was returned without being processed (except at Kalemba Rockshelter; see below).
3) Either the petrous portion of the temporal bone or a tooth or tooth roots were chosen in order to maximize chances of aDNA preservation; appropriate protections were used to minimize contamination. The choice of sample also sought to minimise impacts on osteological collections, for example by choosing samples with an antimere (opposite-side pair), samples that were already fragmentary or isolated from the jaw, and/or samples that would be less useful for morphological analyses due to fragmentation or wear.
4) Digital and photographic databases were created during skeletal inventories and sampling, and were shared with the curating museums.

Table S1 lists the 6 samples from sites in this study that produced usable aDNA. This table also includes 25 samples for which aDNA extraction failed, and 7 samples that were collected but not sampled and were returned to curating institutions intact. The 25 unsuccessful samples came from Fingira (1), Hora (5), Mazinga 1 (5), and Mtuzi (5) in Malawi; from Kirumi Isumbirira (1), Kisese II Rockshelter (1), and Mlambalasi Rockshelter (1) in Tanzania; and from Kalemba Rockshelter (4), Leopard's Hill Cave (1), and Mumbwa Hills Cave (1) in Zambia. Full records of sampling activity at the NMK, NMT, and Livingstone Museum, including at other eastern African Later Stone Age (LSA) sites not presented in the present study (see [2]), have been deposited at these institutions and can be obtained by contacting the curators.

Previously published and reanalysed individuals with increased coverage are from Mota Cave in Ethiopia[55] (I5950; for this individual, also known as Bayira[56], we report ~26x shotgun coverage, allowing reliable calling of diploid genotypes); White Rock Point in Kenya[2] (I8930); Gishimangeda Cave in Tanzania[2] (I13763, I13982, I13983); Chencherere II (I4421, I4422), Fingira (I4426, I4427, I4468), and Hora 1 (I2967) in Malawi[19]; and Shum Laka in Cameroon[17] (I10871, I10872, I10873, I10874).

<u>Sample return and curation of derived products</u>

Skeletal tissue samples exported from Kenya were repatriated to the NMK in October 2017 and June 2019. While no intact tissue samples remain outside the country, remaining powder, DNA extracts, and libraries remain under curation at the Reich Laboratory at Harvard University as agreed upon with the NMK Head of Earth Sciences. All skeletal tissue samples exported from

Tanzania were repatriated to the NMT in May 2019, in keeping with a Memorandum of Agreement (MOA), which allows for curation of remaining powder, DNA extracts, and libraries in the Reich Laboratory. All skeletal tissue samples from the Livingstone Museum were repatriated to that institution in June 2018, except for tissue remaining after radiocarbon dating, which was repatriated in June 2019. No intact tissue samples remain outside of Malawi, and remaining powder, DNA extracts, and libraries remain under curation at the Reich Laboratory at Harvard University.

Samples: archaeological context and dating
For the six newly reported individuals in this paper, we provide background on archaeological sites and burial contexts. In some cases, latitudes and longitudes are approximate, and detailed locations may be obtained from relevant museums or cultural heritage authorities.

All dates are presented as uncalibrated years before present (bp), and as calibrated years before present (cal BP). Previously-published dates are recalibrated here and rounded to the nearest 5 years, using OxCal version 4.4[57], and employing a uniform prior (U(0,100), as in[58], to model mixture of two curves: IntCal2012/14/2021 1:46:00 AM and SHCal20[60].

Fingira and Hora 1

The background to the Fingira and Hora 1 sites and excavations prior to implementation of the Malawi Ancient Lifeways and Peoples Project (MALAPP) are summarised in Skoglund et al.[61]. The three individuals with new genetic data reported here were recovered during MALAPP excavations in 2016 (Fingira) and 2019 (Kahora 1 and Kahora 2), respectively. The site of Fingira (-10.79° lat, 33.77° long) was first excavated in 1966 by Sandelowsky[62], who recovered remains from at least 15 individuals, only one of which was partially articulated. Skoglund et al.[61] reported termite activity documented by MALAPP during renewed test excavations in 2016, which may explain much of this disassociation. Direct dates on human remains reported in that paper range from ~6100-2400 BP, but the distal phalanx that produced new aDNA reported here was recovered from 10cm below the modern surface. Therefore, a likely age is between ~6100-2400, but it may also be younger.

The Hora 1 site (-11.66° lat. 33.64° long.) was first excavated by Clark in 1950[63], who recovered a ~2.2m sequence of deposits that contained two adult human burials (Hora 1, UCT-242, a male; Hora 2, UCT-243, a female)[64]. Note that the human remains recovered from the sites are numbered the same way as the sites themselves, e.g., the Hora 1 site designates this specific rock shelter on the Hora Mountain, while the Hora 1 and Hora 2 burials designate these specific sets of human remains, each of which can be assigned to clear inhumations. Skoglund et al.[61] obtained a direct collagen age on the Hora 2 burial only, leaving the Hora 1 burial to be dated to a similar time period based on a similar depth of burial, similar surrounding material culture, and genetic similarity. Here, we report a new direct age for the Hora 1 inhumation (8075±35, PSUAMS-5145) that verifies they both date to the early Holocene. This new age is based on enamel carbonate prepared from a right maxillary M2 (see **Supplementary Note 4** for methods)

The two new individuals from the Hora 1 site are named Kahora 1 and Kahora 2, “Little Hora 1” and “Little Hora 2”, in the order in which they were discovered. Kahora 1 and Kahora 2 each

represent a >90% complete infant skeleton, with all recovered bones found within an area <0.5m and with most elements articulated in anatomical position (**Figure S1**). Neither yielded collagen suitable for a direct radiocarbon age from the petrous bones that produced the ancient DNA. The bones were so small and fragile that we opted to proceed with associated ages rather than destroy more skeletal tissue for this study. If desirable, it may be possible in future to attempt to obtain direct ages on bones or on a tooth crown. However, the Kahora 1 individual died at such a young ontogenetic age (birth to 5 months based on dental development and skeletal fusion) that only incisor crowns are available. The Kahora 2 individual has an estimated age at death of 7.5±3 months based on dental development and epiphyseal fusion, and some complete molar crowns are present in the crypt. Because a full bioarchaeological and site report is pending, and because direct dating attempts on the petrous bones failed, we describe the circumstances of deposition and association of other dated materials in additional detail below.

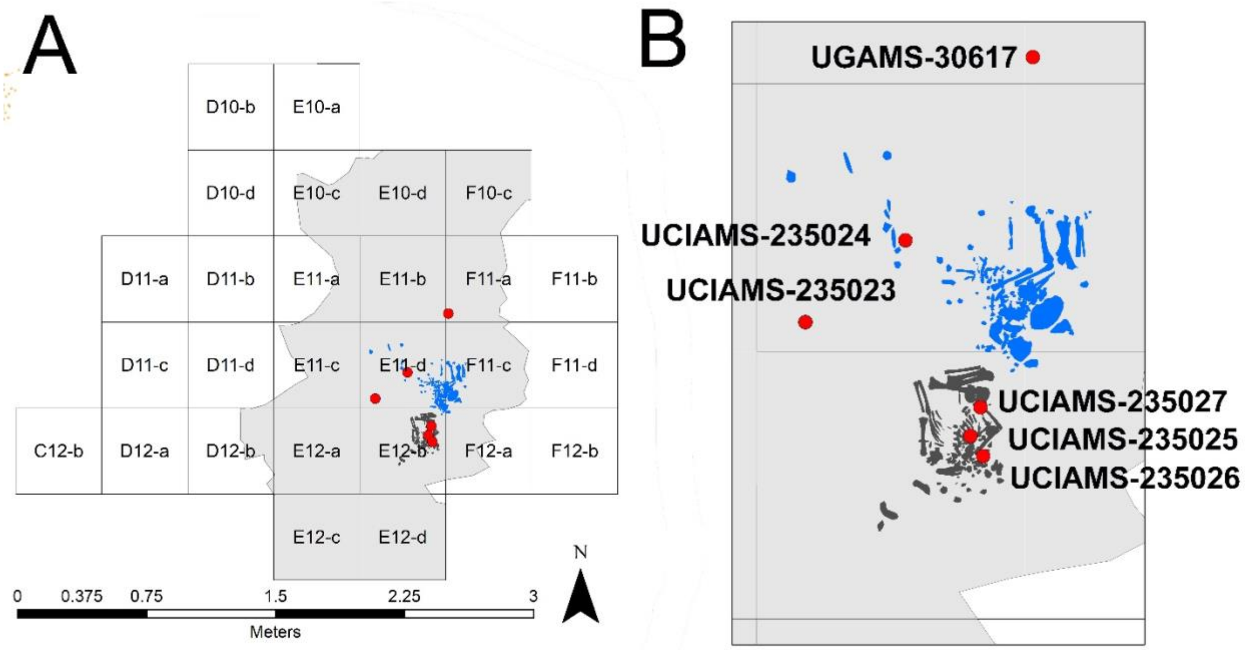

Figure S1. Relationship between Kahora 1 and Kahora 2 infant burials in plan view (**A**), with detail (**B**), showing locations of associated ^{14}C dates and anatomical positioning. Grey area represents extent of the ash feature dated to ~9500 Cal BP, under which both burials were stratified.

The micromorphological samples that were used for the analysis of the Kahora 1 and Kahora 2 infant burials contexts (**Figure S2A-B**) were collected as oriented blocks of intact sediment directly from the archaeological deposits at Hora 1[65]. Petrographic thin sections produced from these block samples were described following the work by Courty et al.[66] and Stoops[67]. Thin sections are currently kept at the Interdisciplinary Centre for Archaeology and the Evolution of Human Behaviour (ICArEHB), where they are used for further analysis. Reproducibility is warranted by long-term storage of slices of the impregnated and hardened micromorphological blocks, so that in future more thin sections may be produced of the same samples.

The archaeological deposits at Hora 1 contain high amounts of ash and other combustion related materials, which are interspersed with angular fragments of granitic bedrock, ranging in size from coarse sand to medium gravel. Ash coatings on bone fragments and other coarse particles indicate that coarse components have frequently been moved somewhat during or shortly after deposition. These coatings readily form in the ashy sediment due to its softness and poor consistency, and may be related to hearth rake-out activities (**Figure S2B**).

The infant skeletons were buried in and covered by combustion residues, creating no visible pit features[68]. Post-depositional disturbance by termites is observed at Kahora 1. The construction of clay-fortified channels by termites can move small (sand-sized) fragments of bone, charcoal, and shell over an estimated distance of up to 2 cm. Larger fragments are unlikely to have been significantly displaced by termites after deposition, and elongate objects remain lying flat *in situ* (**Figure S2A**). Below the level of Kahora 1, including Kahora 2, no termite activity is observed in thin section, but termite activity is still indicated by chunks of clay that could be felt by hand during excavation. The anatomical completeness and articulated state of both sets of remains indicates deposition near the time of death and with little post-depositional disturbance, especially for Kahora 1. Kahora 2 appears to have suffered post-depositional disturbance and dispersal of thoracic elements, leaving the limb bones and cranium intact. The lack of pit features is explicable by the homogenous nature of the surrounding ashy sediments and subsequent blurring of feature boundaries by termites – but without post-depositional movement sufficient to relocate the majority of elements.

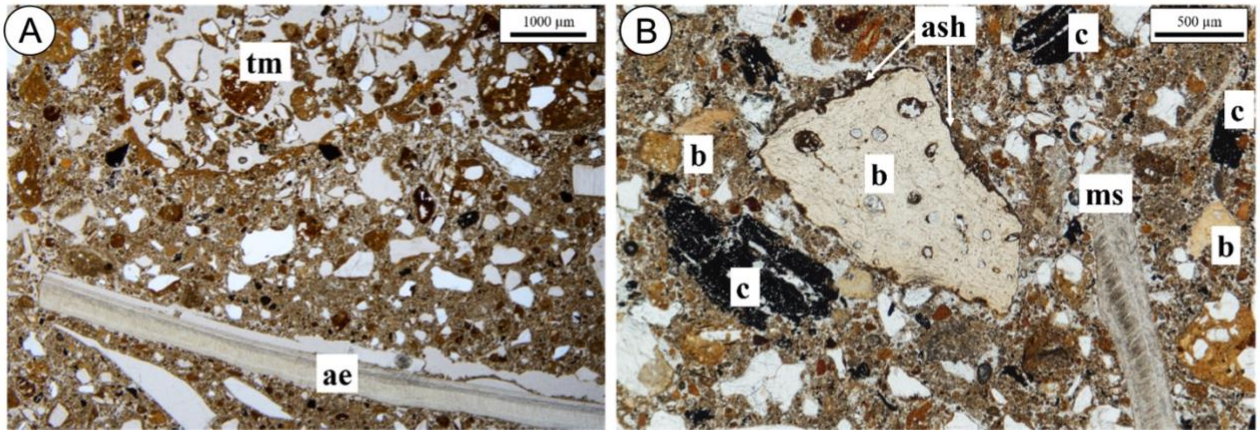

Figure S2. Photomicrographs from the Kahora 1 and Kahora 2 burial contexts. (A) termites (tm) have disturbed the sediment at Kahora 1 by constructing channels lined with clay (tm). Larger fragments are less likely to have been moved significantly, e. g., an ca. 8 mm long avian eggshell (ae) appears in its original, near-horizontal, position only ca. 2 mm below a termite channel. (B) Kahora 2 sediment contains abundant bone fragments (b), here shown in close association with charcoal fragments (c) and mollusk shell (ms). (b). Ash coatings are indicative of syndepositional movement. Sand and very fine gravel appear white. Photomicrographs shown here were taken with plane polarised light at 100x (**2A**) and 40x (**2B**) magnification from locations representative of the infant burial contexts and selected to demonstrate features mentioned in the text.

Both individuals were recovered from deposits stratified below a large, consolidated ash feature (~200 x 100 cm wide by 20 cm thick) dated to ~9500 cal BP based on charcoal (8520±25,

UGAMS-30617). This provides the minimum possible age of deposition for both burials, but associated and well-stratified dates on land snail shell indicate that a more likely age is in the terminal Pleistocene, with a range of ~16,000-14,000 for Kahora 1 and 17,000-16,000 for Kahora 2 (**Figure S3**).

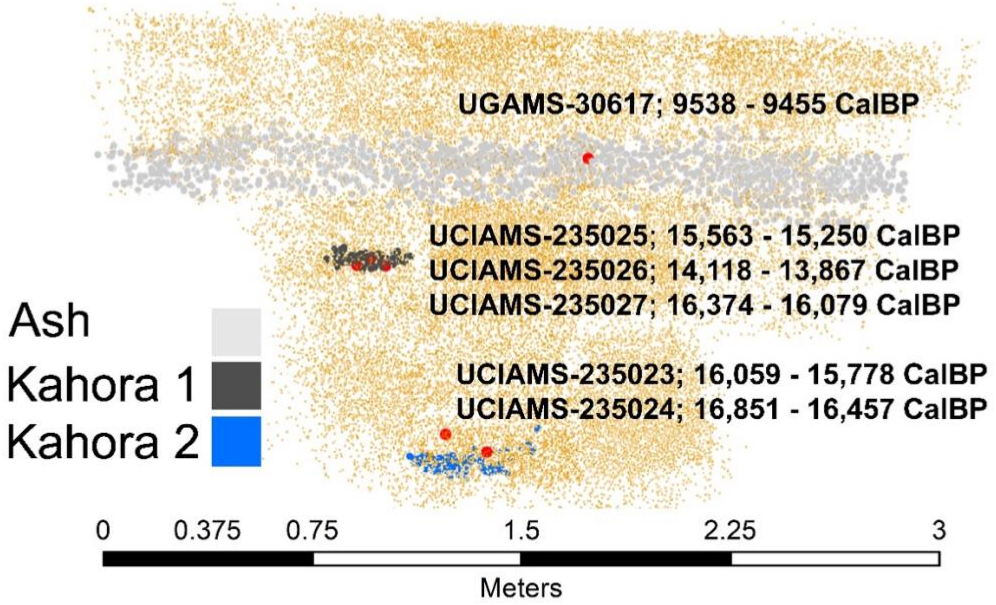

Figure S3. View west showing the large ash mound (light grey) in relation to the total excavated area (brown), the Kahora 1 and Kahora 2 burials, with associated ^{14}C samples.

Kalemba Rockshelter

Kalemba Rockshelter is located in the southeastern Chipwete Valley in Chadiza District, Eastern Province, Zambia, at approximately -14.12° lat, 32.50° long and 1200 meters above sea level (m asl). Among the largest known rockshelters in Zambia, Kalemba is recognised for its deep archaeological sequence spanning the Middle Stone Age (MSA) to LSA transition through the Iron Age, as well as for rock art: a nearly 12-meter-long display of white, mainly anthropomorphic paintings, overlying earlier red geometric traces. Phillipson[69] suggests at least some of the white paintings may be of cattle, indicating a potentially late date.

Excavations at Kalemba Rockshelter were initiated by Phillipson in 1971, as part of a broader study of Late Pleistocene through Holocene archaeology of the region, including at nearby Makwe and Thandwe Rockshelters[70,71]. Together, these shelters enabled Phillipson to outline a nearly 40,000-year regional archaeological sequence. Recent faunal and stable isotope analyses have shed new light on environmental conditions and subsistence strategies at Kalemba[72].

Phillipson excavated 40.2 m^2 of the shelter and reached a maximum depth (in just 1 m^2) of 4.3 m. The deposit was divided into 47 stratigraphic layers grouped into 13 horizons. A series of radiocarbon dates enabled these to be further grouped into phases (**Table S13)**. The MSA phase in Horizon G (levels 47-46) dates to greater than 37,000 BP and is followed by a hiatus. The MSA-LSA transition in Horizons H, I, J, and the bottom of K (levels 45-30) dates to ~35-25 ka

and is followed by another hiatus until after the Last Glacial Maximum. The next phase is characterised by the LSA "Nachikufan I" industry (described below) from the top of Horizon K through Horizon N (levels 29-15) and arguably dating to ~21-15 ka (details in[73]). In the Holocene, this is followed by the "Makwe Industry" in Horizons O, P, and Q (levels 14-6), dating to ~9-5 ka. At the top of the sequence are Early and Later Iron Age deposits in Horizons R and S (levels 5-1); the only date is a recent one from the uppermost deposit.

Table S13. Published dates from Kalemba Rockshelter[70,71]
For dates with distinct uncertainties (e.g., +2000, -1000), the larger value was used as both +/-.

Level	Horizon	Square	Material	Lab No.	Uncal bp	Cal BP	Industry
2	S	L/M 16	Beans from pot	N-1389	115±70	285-0	Later Iron Age
6	Q top	I 14	Charcoal	N-1387	4480±90	5320-4860	Makwe
8	Q bottom	I 15	Charcoal	N-1386	5040±110	6100-5485	Makwe
10	P lower	H 15	Charcoal	N-1385	7030±105	8020-7625	Makwe
11/12	O upper	H/I 15	Charcoal	N-1388	6810±105	7915-7435	Makwe
12	O mid	H 15	Bone apatite	GX-2770	7915±300	9525-8175	Makwe
15	N mid	H 15	Bone apatite	GX-2769	15330±1100	21870-16055	Nachikufan
19	M mid	H 16/17	Bone apatite	GX-2768	26300 +1500, -1200	34510-27780	Nachikufan
24	L mid	H 16/17	Bone apatite	GX-2767	>31000	N/A	Nachikufan
27	K top	H 17	Bone apatite	GX-2766	14800±1000	20770-15495	Nachikufan
30	K lower	H 16/17	Bone apatite	GX-2611	24420 +2000, -1000	35225-25175	MSA-LSA transition
40	H top	H/I 17	Bone apatite	GX-2610	24600 +2000, -1000	35340-25290	MSA-LSA transition
46/47	G bottom	H 16	Bone apatite	GX-2609	>37000	N/A	MSA

The Kalemba archaeological sequence documents a series of technological changes over some forty millennia of hunting and gathering. The first is a transition from an MSA (Mode 3) industry with unifacial points and scrapers, to an LSA Mode 5 industry characterised by a slight reduction in artifact size and increase in blade-based tools. The next is a post-LGM appearance of the Nachikufan industry, defined by Clark[74] based on Zambian sites west of Kalemba. According to Kusimba[75] this is "characterised by pointed backed bladelets, some backed crescent forms and scrapers, bone points, bored stones, and ground stone axes." This is followed by the Holocene Makwe Industry, defined by Phillipson[71] as an increasingly microlithic industry where scrapers, backed flakes, and backed bladelets were replaced by geometric microliths and crescents. It is also associated with ground stone axes, and with an increased frequency of bone tools and bone and shell beads, although beads are more common at the Makwe site than at Kalemba.

The Iron Age deposits are relatively thin and contain limited diagnostic pottery: a single Early Iron Age potsherd (among other less diagnostic sherds) in Horizon R, and 59 Later Iron Age sherds in Horizon S. While a few pieces of worked iron were found in near-surface deposits,

there was no evidence for ironworking activities. The faunal remains in the Holocene deposits are characterised by a shift to smaller game as well as land snail shell exploitation; there is no evidence for livestock.

The remains of five humans were uncovered at Kalemba (**Table S14)**, the most ancient being a single isolated tooth in Horizon L, associated with the terminal Pleistocene Nachikufan; this was designated SK1 by de Villiers[76] in her osteological analysis. The other human remains come from burials in the middle Holocene Makwe Industry phase: in Horizon O, one double burial of a young adult female and an infant (SK2, SK3) and one single burial of a child (SK4), and in Horizon Q a single burial of an adult female (SK5). SK5 was initially reported as a probable male based on osteology[76], but was later reported as female[77], which is also the genetic sex reported here.

The Kalemba burials appear to be secondary and were principally skulls and cervical vertebrae, with postcranial remains nearly absent. Phillipson[71] interpreted the skulls as broken prior to burial; in one case (SK5), postcranial bones were fragmented and burnt prior to interment. Phillipson noted that the SK5 remains were interred in a small burial pit (c. 20 cm diameter) lined with pelvic and skull remains, and with the two hemi-mandibles marking each end of the pit, and other postcranial remains filling the space in between. Phillipson[71] wrote, "One is left with the view that considerable importance was attached to the orderly disposal of the dead, but that burial was delayed until the corpses had become fragmented, whether by natural causes or by artificial means. It is hard to avoid the conclusion that the separate burial of fragmented heads at Kalemba may have been associated with some form of ritual dismemberment, perhaps involving the removal of the brain."

Human remains were also found at the archaeologically related Makwe and Thandwe Rockshelters[76], but these could not be located in the Livingstone Museum in 2017. The Kalemba skeletal remains were in their original paper context bags, and individuals were split across several bags. Some human remains were commingled in "miscellaneous" bags, making it difficult to differentiate individuals. For this reason, we accidentally sampled two specimens from the same individual (SK5). We also sampled SK2, SK3, and SK4, but did not sample SK1 which is represented by a single tooth crown. Only SK5 (sample number KLB.03.01) produced usable ancient DNA.

SK5 was previously dated to ~6.1-4.9 ka via charcoal (N-1387, N-1386) found in the same horizon (Q), but two levels apart and in different grid squares from each other and from the burial located in Level 7. A new date on SK5 of ~5.3-5 ka (4480±20 bp, PSUAMS 4764) is consistent with and refines this range.

Table S14. Human remains and samples collected from Kalemba Rockshelter (see Table S1)

aDNA sample ID	Skeleton	Square/ Level/ Horizon	Associated industry	Description, based on de Villiers[76]	Samples collected
KLB.03.01, KLB. 04.01	SK5	K10/7/Q	Makwe	Fragmented but near complete cranium and mandible, and fragmentary axial and	KLB.03.01 is a sampled petrous (S10726), and KLB.04.01 is a sampled tooth (S10833), at the time thought to be from a different

				appendicular postcranial remains. Fully adult individual, possibly male. Shallow grave within Horizon Q; small pit with apparently secondary burial.	individual due to commingling, leading to accidental double-sampling of the same individual.
KLB.02.01	SK4	J15/13/O	Makwe	Fragmented cranium and single permanent tooth crown; juvenile estimated to be 7-8 years old. Placed under a stone slab.	KLB.02.01 is a sampled petrous (S10725).
KLB.05.01	SK3	I11/16/O	Makwe	Infantile remains, including fragmented cranium with 2 teeth, 2 ribs, 4 vertebrae; interred in the same grave as SK2.	KLB.05.01 is a sampled cranial fragment (S10086).
KLB.01.01; KLB.01.02	SK2	I11/16/O	Makwe	Fragmented but near complete cranium and mandible, and cervical vertebrae, young adult female. Shallow grave dug into Horizon L from Horizon O.	KLB.01.01 is a sampled petrous (S10724); KLB.01.02 is a canine that was collected but later not sampled and returned intact.
Not sampled	SK1	I17/?/L	Nachikufan	Isolated tooth; crown of lateral incisor.	No sample collected.

Kisese II Rockshelter

Kisese II (-4.49° lat, 35.81° long, 1287±3 m asl) is a painted rockshelter within the UNESCO World Heritage Kondoa Rock-Art Sites, a region that contains the richest record of hunter-gatherer and pastoralist rock art in eastern Africa[78–81]. The site consists of an east-facing overhang on one of three large (>100 m^3) adjoining boulders ~200 m below the escarpment of the Irangi Hills, the eastern margin of the Gregory Rift Valley. These boulders and the shelter beneath them are a locally prominent and readily visible landscape feature. Mean annual precipitation in the area today is ~850 mm.

Archaeological investigations at the site began in 1935 when Louis and Mary Leakey visited it to study and document the rock paintings[81–84], which include humans, animals (especially giraffe), and geometric figures executed in red and white. With the aim of dating the rock art, the Leakeys excavated a ~5.3 m^2 trial trench to a depth of ~4 m in 1951[84] but no details were published. At their invitation, Raymond Inskeep expanded the initial trial trench in 1956, excavating 21.3 m^2 in ~10-inch spits through generally silty sediments to a depth of ~6 m. Inskeep's excavations successfully located *in situ* painted, exfoliated slabs of the shelter wall, recovered a large sample of well preserved and taxonomically diverse fossil fauna, thousands of ostrich egg-shell beads, and >5,900 lithic artifacts. Only a very brief summary of the excavation was published[85]. Fauna and human remains from Inskeep's collections were sent to what is now the National Museums of Kenya in Nairobi following excavation, whereas the remainder of the material was sent to what is now the Tanzanian National Museum in Dar es Salaam.

A new project was initiated in 2011 to reconstruct the Kisese II site's chronology, document the nature of archaeological and environmental change through the sequence, and assess the nature of the site's human remains[86–91]. Thus far, this work resulted in a sequence of calibrated radiocarbon dates that generally increased in age with depth (**Table S15**), demonstrating that parts of the deposit date beyond the radiocarbon ceiling *ca*. 45 ka and that additional undated archaeological sediments underlie the deposits excavated by Inskeep. Renewed excavations at the site by the Kondoa Archaeological Research Heritage Project (KARHP) began in 2017 and follow a community archaeology model[92].

Kisese II was a place on the landscape intermittently occupied for at least 45,000 years, based on the radiocarbon dates on ostrich eggshell fragments from the upper portion of the stratigraphic sequence[90]. Although large portions of the material originally excavated by Inskeep have since been lost, the available collections, in combination with Inskeep's archives and new excavations, enable a preliminary understanding of behavioural change over time at Kisese II. Some patterns can be seen in the lithic technology, predominantly quartz throughout the sequence, in which prepared centripetal technologies are more common in the lower levels (Spit XIII and below) and almost entirely disappear in strata dating to after the Last Glacial Maximum[88]. Backed elements, especially crescents, are more abundant in upper levels (below Spit XIII). Grindstones, ochre, and evidence of ostrich eggshell bead production is present throughout much of the sequence[90]. In addition to a turnover in the kinds of tools produced and ways of making them, ostrich eggshell beads show change over time within a single technology, declining in size presumably as a result of a shift towards a preference for smaller beads, perhaps indicating new ways into which they were incorporated onto bodies or objects[88].

Our interpretation of Inskeep's excavations suggest an increase in occupation intensity during the Last Glacial Maximum, but continued refinement of the chronology and archaeological data are required to further explore this. The rock art at Kisese II remains of unknown age, though specialists note the presence of two distinct genres (red fine line and white[79,81]). Holocene occupants appear to have used lithic, ceramic, and iron technologies, to have incorporated at least some domesticated animals into their diet, and to have at least occasionally had access to stone raw materials (obsidian) that suggest social connections to groups in regions outside Kondoa[90].

Table S15. Radiocarbon dates from Kisese II. All dates on the carbonate fraction of ostrich eggshell, calibrated using the IntCal20 calibration curve with Calib v.8.2 software[93] using a 50%:50% combined IntCal20/SHCal20 calibration curve following the recommendations of Reimer et al.[59] and Hogg et al.[60] reported at 95.4% probability. * and ** indicate samples with overlapping $\delta^{13}C$ values and calibrated radiocarbon dates, which could potentially derive from the same ostrich eggshell. ***Date on Burial 4/5/6 of Laird et al.[86]; precise stratigraphic position unknown. UBA are University of Belfast AMS dates on unburnt OES. NPL are National Public Laboratory conventional ^{14}C dates on burnt OES[94]. PSUAMS is an AMS date on unburnt bone. NPL-38 was calibrated using the higher of the two variance values.

Spit	Laboratory code	^{14}C yr BP ($\pm 1\sigma$)	calBP (95.4%)
I	UBA-27427*	3,870 ± 30	4,407 – 4,153

	UBA-27428*	3,840 ± 30	4,403 – 4,092
II	UBA-27430	3,770 ± 20	4,231 – 3,987
	UBA-27429	14,830 ± 60	18,254 – 17,949
III	UBA-27431	14,270 ± 60	17,468 – 17,083
	UBA-27432	14,020 ± 60	17,323 – 16,835
IV	NPL-35	14,760 ± 200	18,607 – 17,394
V	UBA-27433	15,410 ± 70	18,847 – 18,338
	UBA-27434	9,320 ± 40	10,649 – 10,300
	UBA-34477	14,880 ± 60	18,276 – 18,007
VII	NPL-36	10,720 ±130	12,967 – 12,103
	UBA-34478	38,040 ± 400	42,591 – 41,934
IX	NPL-37	18,190 ± 310	22,814 – 21,160
X	UBA-34479	19,480 ± 80	23,760 – 23,179
XI	UBA-34480	30,800 ± 220	35,577 – 34,616
XII	UBA-27435**	30,620 ± 280	35,494 – 34,451
	UBA-27436	30,070 ± 250	35,122 – 34,110
XIV	NPL-38	31,480 +1,640/-1,350	39,738 – 32,152
XV	UBA-27437	30,930 ± 300	35,975 – 34,636
	UBA-27438**	31,340 ± 290	36,287– 35,138
XVIII	UBA-34481	34,380 ± 290	40,348 – 38,934
XIX	UBA-34482	27,790 ± 140	31,976 – 31,303
	UBA-34483	36,740 ± 680	42,317 – 40,540
	UBA-27439	33,420 ± 380	39,264 – 37,074
	UBA-27440	41,200 ± 1,000	45,535 – 42,724
XX	UBA-34484	40,600 ± 1,000	45,015 – 42,482
XXI	UBA-34485	22,200 ± 120	26,906 – 26,017
	UBA-27441	15,820 ± 60	19,209 – 18,900

	UBA-27442	41,300 ± 1,000	45,627 – 42,769
N/A***	PSUAMS-4718	6,210 ± 30	7,240 – 6,985

All human remains from Kisese II were excavated by Inskeep in 1956. The human remains are currently housed at the National Museum of Kenya in Nairobi, but the Kisese II artifacts, fauna, and select human skeletal elements were returned to the National Museum of Tanzania in Dar es Salaam. Photographs from the original excavation show the presence of adult long bones, but these elements were not located in the current collections. The Kisese II burials consist of a minimum of six individuals including two adults and four juveniles[86]. Two of the juveniles were sampled for this study-KNM-KX 4/5/6 and KNM-KX 7/8 (**Table S16**). The ages of these individuals were estimated based on dental eruption and the unfused basiocciput and humeri. Sex was not estimated as both individuals are sub-adults.

Dating for most of the human burials is uncertain. Handwritten notes and photographs suggest human remains may be as deep as spit XIV (39,920-33,010 calBP)[90]. Ostrich eggshell beads associated with two of the burials suggest a younger date[89]. The petrous portion of one of the juveniles, KNM-KX 4/5/6, was AMS radiocarbon dated to 6210±30 uncal bp and has a calibrated age range of 7,240-6,985 cal yr BP at a 95% confidence interval (**Table S3**). This is the same petrous portion that yielded DNA.

Table S16. Human remains from Kisese II Rockshelter and samples collected (see Table S1)

aDNA sample ID	Skeleton	Notes	Samples collected
KX5.01, KX5.02	KNM-KX 4/5/6	Juvenile estimated between 3-5 years of age. Consists of a fragmented cranium, petrous, mandible, maxilla, basiocciput, and select postcranial remains including the scapula, clavicle, ulna, radius, and humerus.	KX5.01 is a sampled petrous (S8821), KX5.02 is a tooth that was collected but later not sampled and was returned intact.
KX8.01, KX8.02	KNM-KX 7/8	Juvenile estimated to be 2 years ± 8 months in age. Fragmented cranium, mandible, maxilla, petrous, and basiocciput along with a scapula and humerus.	KX8.01 is a sampled petrous (S8811), KX8.02 is a tooth that was collected but later not sampled and was returned intact.

Mlambalasi Rockshelter

Mlambalasi Rockshelter is part of a granitic inselberg located west of the city of Iringa, Tanzania at approximately -7.59 lat, 35.50 long, and 1029 m asl. The rockshelter is best known as the hideout of Chief Mkwawa, paramount chief of the Hehe, and is the place where he ultimately killed himself in 1898 to avoid capture by German colonial authorities[95]. The site is now the location of a National Monument and Mkwawa’s tomb (designated HwJf-01). The main rockshelter (HwJf-02), located a couple of meters up the escarpment from the monument,

preserves a record spanning the terminal Pleistocene LSA to the historic period as well as a rock art panel[96].

Mlambalasi was initially excavated in 2002 by a team from the National Museum in Dar es Salaam led by Dr. Paul Msemwa who dug a 2x1m^2 trench under the shelter overhang to a depth of approximately 60cm. The excavators brought a representative sample of the finds back to the National Museum, but the site was not given a SASES number. Unaware of the previous excavation, the Iringa Region Archaeological Project (IRAP) excavated two additional test units in 2006 that by chance did not overlap with previous excavations. In 2010, IRAP excavated a 2x3m^2 trench to a maximum depth of 110cm to establish context for previously excavated finds and document the full stratigraphic sequence.

The site's stratigraphy is complicated by its long history of occupation as well as taphonomic factors including termite activity and recent trampling damage from use as an animal enclosure. LSA levels were characterised by abundant lithics, OES beads, and faunal remains, many of which were coated in a calcium carbonate cement. The overlying Iron Age/historic deposits were marked by highly visible changes in stratigraphy associated with iron smelting, as well as the addition of iron slag, tuyère fragments, ceramics, and glass/plastic beads. Fourteen radiocarbon dates on charcoal, snail shell and OES beads establish a sequence spanning approximately ~20 ka to the last several hundred years inside the rockshelter[96]. MSA artifacts present on the slope outside the rockshelter suggest the occupation of the rockshelter extends even further back.

Human remains were recovered from all three field seasons and represent at least three individuals[96,97]. Two commingled individuals are associated with the older LSA occupation and consist of one adult (B-1) and one juvenile (B-2), with another adult potentially represented. The partial skeleton of another adult, Burial 3, was recovered from an Iron Age horizon a few meters away. The remains are exceptionally fragmentary, with poor preservation likely compounded by the Iron Age smelting activity and recent trampling. All individuals are curated at the National Museum in Dar es Salaam. We collected aDNA samples from the two best preserved individuals, one from the LSA (B-1) and one from the Iron Age (B-3), and obtained aDNA from the former (**Table S17**).

The B-1 individual was in a primary *in situ* burial toward the back of the rockshelter approximately 70-90 cm below the modern ground surface. The sample failed to produce a radiocarbon date, consistent with previous attempts to directly date human remains from the site[97]. However, an OES bead near the B-1 individual's right wrist was directly dated to ~17.3-17 ka (14115±55 bp, OxA-27621). Two additional beads from within the burial feature have been dated to ~17.5-17.1 ka (14275±55 bp, OxA-27623) and ~20.3-19.9 ka (16690±65 bp, OxA-27624). Other radiocarbon dates on charcoal and snail shells, as well as associated large LSA artifacts, are also consistent with a terminal Pleistocene age for the burial (the full list of dates is provided in Biittner et al.[96]). In addition to being one of few terminal Pleistocene skeletons from eastern Africa, this individual is notable for their small stature and body size, as well as indications of advanced dental disease[96,97].

Table S17. Human remains from Mlambalasi Rockshelter and samples collected (see also Table S1)

aDNA sample ID	Skeleton	Notes	Samples collected
2010.09.24<br>2010.09.71	HwJf-02 B-1	Fragmentary but largely complete skeleton of a middle-aged adult. Bioarchaeological sex could not be estimated, but genetic sex is female. The skeleton was in a primary burial toward the back of the rockshelter.	2010.09.24 is a sampled petrous (S13976). 2010.09.71 is an upper canine that was collected but later not sampled and was returned intact.
2002.03<br>2010.36	HwJf-02 B-3	Fragmentary skull and upper body of an adult, possibly female. Burial was located near an Iron Age smelting furnace near the entrance of the rockshelter.	2003.03 is a sampled molar root in mandible fragment (S14021). 2010.36 was a cranial fragment that was collected but later not sampled and was returned intact.

Supplementary Note 4. Radiocarbon dating procedures

Radiocarbon dating of bone collagen

The six bone samples (five petrous bones and one phalanx) that produced readable aDNA were submitted to the Pennsylvania State University (PSU) Radiocarbon Laboratory for radiocarbon dating via accelerator mass spectrometry (AMS) (**Table S3**). Possible consolidant and adhesive contamination was addressed by pre-emptively sonicating all bone samples in successive washes of ACS grade methanol, acetone, and dichloromethane for 30 minutes each at room temperature, followed by three washes in 18.2 MΩ/cm water to rinse. Samples (200–400 mg) were demineralised for 24–36 h in 0.5N HCl at 5 °C followed by a brief (<1 h) alkali bath in 0.1N NaOH at room temperature to remove humates. The residue was rinsed to neutrality in multiple changes of 18.2 MΩ/cm H_2O, and then gelatinised for 12 h at 60 °C in 0.01N HCl. The resulting gelatin was lyophilised and weighed to determine percent yield as a first evaluation of the degree of bone collagen preservation. Better preserved gelatin samples were rehydrated and the solution was pipetted into pre-cleaned Centriprep[98] ultrafilters (retaining >30 kDa molecular weight gelatin) and centrifuged 3 times for 20 min, diluted with 18.2 MΩ/cm H_2O and centrifuged 3 more times for 20 min to desalt the solution.

Less well-preserved gelatin samples were processed by a modified XAD technique[99]. The sample gelatin was hydrolyzed in 2 mL 6 N HCl for 24 h at 110°C. Supelco ENVI-Chrom® SPE (Solid Phase Extraction; SigmaeAldrich) columns were prepped, with a 0.45 mm Millex Durapore filter attached, by equilibrating the medium with 50 mL 6 N HCl. 2 mL collagen hydrolyzate as HCl was pipetted onto the SPE column and driven with an additional 10 mL 6 N HCl dropwise with the syringe into a 20 mm culture tube. The hydrolyzate was finally dried into a viscous syrup by passing UHP N2 gas over the sample heated at 50°C for ~12 h.

For the two bone samples that were subject to radiocarbon dating, carbon and nitrogen concentrations and stable isotope ratios of the ultrafiltered collagen/XAD amino acid samples were measured at the Yale Analytical and Stable Isotope Center with a Costech elemental analyser (ECS 4010) and Thermo DeltaPlus isotope ratio mass spectrometer. Sample quality was evaluated by % crude gelatin yield (strictly speaking the acid-insoluble fraction, potentially also comprising non-collagenous components), %C, %N and C/N ratios before AMS ^{14}C dating. C/N ratios for all samples fell between 3.3 and 3.4, indicating good collagen preservation[100,101]. Samples (~2.1 mg) were then combusted for 3 h at 900°C in vacuum-sealed quartz tubes with CuO and Ag wires. Sample CO_2 was reduced to graphite at 550°C using H_2 and a Fe catalyst, with reaction water drawn off with $Mg(ClO_4)_2$[102]. Graphite samples were pressed into targets in Al boats and loaded on a target wheel with primary and secondary standards and blanks. ^{14}C measurements were made at PSU on a modified National Electronics Corporation compact spectrometer with a 0.5 MV accelerator (NEC 1.5SDH-1). The ^{14}C ages were corrected for fractionation with $\delta^{13}C$ values measured on the AMS (i.e., $^{13}C^+/^{12}C^+$)[103] and normalised using OXII (oxalic acid) with blank corrections made using samples of Pleistocene whale bone (Beaufort whale, >48,000 ^{14}C BP), late Holocene bison bone (~1,850 ^{14}C BP), and late 1800s CE cow bone.

Four samples failed to produce sufficient collagen for a date. For the two successful samples, radiocarbon ages were calibrated using OxCal version 4.4[57], employing a uniform prior

(U(0,100)) that allows the program to model an unspecified mixture of two curves: IntCal20[59] and SHCal20[60]. This approach may help to account for the effects of the Intertropical Convergence Zone (ITCZ). Reservoir corrections were not applied based on stable carbon and nitrogen isotopes and the absence of marine or aquatic resources in associated archaeological deposits.

Radiocarbon dating of dental enamel

For the individual from Hora 1 (I2966), a radiocarbon date was generated from dental enamel carbonate. Before destructive analysis, the tooth was photographed and scanned using µCT. Enamel pretreatment methods are described in detail in Jones et al.[104]. Enamel apatite sample preparation and purification at the University of Illinois Environmental Isotope Paleobiogeochemistry Laboratory-EIPL is based on Krueger's[105] "Vacuum Milling" technique for bone apatite, dentine and enamel carbonate dating[106–108], with modifications of the procedure to reduce recrystallisation and isotopic exchange during chemical purification. Dentine and cementum were separated from enamel using a Kupa® Mani-Pro KP-5000 handpiece drill with carbide dental bits, at or near the lowest speed setting in order to minimise frictional heating. Samples of cleaned enamel fragments were sonicated, freeze-dried and finely ground using an agate mortar.

Powdered enamel was placed in pre-weighed 50 ml plastic centrifuge tubes with screw-top lids removed. Samples were treated with 25 ml 2.63% NaClO (sodium hypochlorite) for ~20 hours to remove organic matter, then rinsed 5x with distilled H_2O, then reacted with 25 ml 0.1 M acetic acid (~0.1 ml per mg), to remove adsorbed and diagenetic carbonate[109]. Treatment with 1.0 M acetic acid, may cause recrystallisation, locking in diagenetic carbonate. Samples were reacted under vacuum and briefly restored to atmospheric pressure every ~15-30 minutes using CO_2-free N_2 drawn from the headspace of a liquid N_2 dewar. Samples initially reacted vigorously under vacuum, so pressure was reduced gradually. As reaction slowed, tubes were tapped to release trapped CO_2. Treatment continued until effervescence ceased when tapped vigorously, usually after ~3-4 hours. Samples were rinsed 5x in distilled H_2O and freeze dried with lids placed loosely on, but not sealing, the tubes. When dry, tubes were returned to atmospheric pressure with CO_2-free N_2, and caps immediately closed tightly, weighed to calculate pretreatment weight loss, and sealed tightly with wax parafilm to prevent atmospheric CO_2 exchange before radiocarbon analysis.

Supplementary Note 5. Phenotype-associated SNPs and data authenticity

Phenotype-associated SNPs

We examined genotypes at SNPs known to be associated with lactase persistence (5), sickle cell trait, and the Duffy antigen (**Supplementary Table S5**). Due to low sequencing coverage, most individuals and SNPs have limited or no data, but some of the higher-coverage individuals provided multiple reads per locus. Overall, we found almost no copies of derived alleles in any of the individuals, with the only exceptions being rs2814778 (DARC/Duffy negative) for the four previously published individuals from Shum Laka in Cameroon (all observed alleles derived). Across the seven SNPs, the total number of reads from all individuals ranged from 46 to > 300, with 4-11 individuals represented by at least four reads. Thus, while we remain unsure of many of the individuals' genotypes, our (limited) results do not provide any positive evidence for the presence of the tested alleles among ancient eastern, south-central, and southern African foragers, and only one example for west-central African foragers.

Authenticity and contamination

We used a combination of approaches to assess the authenticity of the aDNA data and evaluate possible contamination. All of the methods are more reliable and informative for higher-coverage data, so we provide a caveat that we cannot evaluate the low-coverage samples as carefully as those with high coverage, but the results of our study are also less reliant on conclusions drawn from those samples.

1. Molecular attributes

We observed characteristic ancient DNA deamination damage in all libraries (**Supplementary Table S2**), with damage rates in the last base position of molecules mapped to the nuclear genome falling within expected ranges[110–113]. For the Shum Laka individuals, whose DNA was known to be very well preserved[17], damage ranged from 0.19-0.26 for double-stranded non-UDG-treated libraries, 0.06-0.10 for double-stranded UDG-treated, 0.11-0.14 for single-stranded non-UDG-treated, and 0.07-0.09 for single-stranded UDG-treated. All libraries for other individuals were UDG-treated and had damage rates between 0.03-0.19 for double-stranded preparation and 0.20-0.44 for single-stranded preparation. As expected, all libraries also had short average DNA fragment lengths (maximum of 67 bases).

2. Sex chromosome ratio

For each library, we computed a sex chromosome ratio of the form Y/(X+Y), where X and Y are the numbers of sequences aligning to the X and Y chromosomes (**Extended Data Figure 1A**). Females are expected to have a ratio close to zero, and males are expected to have a substantially higher ratio (closer to 0.4 given the relative number of X chromosome and Y chromosome SNPs that are targeted through in-solution enrichment). Intermediate values suggest contamination, with the limitation that in order to be detectable it must be from an individual of the opposite sex. In our analysis, we omitted libraries with very low coverage (< 2500 SNPs), which are marked as "U" (unknown sex) in **Supplementary Table S2**. Among females, one individual (I13763) had ratios of ~0.03-0.1 across libraries, indicative of modest contamination. All other libraries had point estimates of < 0.05, with 95% confidence intervals consistent with ratios of 0.015 or lower. For males, not all libraries and individuals converge to a single ratio, due to differences in preservation or other factors. However, we found that all confidence intervals overlapped a

narrow range between 0.382 and 0.436, with no visible outliers on the low end. Thus, this method identifies only I13763 as likely having a modest amount of contamination

3. Apparent heterozygosity for mitochondrial DNA
Next, we searched for apparent heterozygous sites in mtDNA, which are indicative of contamination (from an individual with a different haplogroup). Results are given in **Supplementary Table S2** in the form of a 95% confidence interval (CI) for the matching rate, where higher values (maximum 1) imply less contamination. As with all contamination estimators, the utility of the method is greater for higher-coverage data (unreliable below ~2x in our experience). There can also be a discordance between mtDNA and nuclear contamination rates if the endogenous and contaminant DNA have different ratios of mt/nuclear fragments. We observe minimal mismatching (on the order of a few percent at most; maximum ~5% for I19528) for libraries with > 5x mtDNA coverage. Two individuals showed modest mismatching at coverages between 2x and 5x. First, I19529 (Hora 1, 4.3x mtDNA coverage) had a match rate CI of 85.9-95.1%. Second, I10726 (Kalemba) had variable match rates across six libraries prepared from the same DNA extract. However, there was a clear trend for higher match rates in the higher-coverage libraries (i.e., with the more reliable estimates), with CIs of 88.3-96.0%, 87.0-94.5%, and 96.2-98.7% for the three highest (**Supplementary Table S2**). We also did not find any contamination signals for these two individuals using our other approaches. Thus, while the results could warrant some slight extra caution, our overall evaluation was that they are not significantly contaminated.

4. Apparent heterozygosity for male X chromosomes
This method is similar to the previous one, but it is applicable for males only and generally requires fairly high coverage. We obtained contamination estimates for five individuals (one newly reported); the maximum point estimate for any library was 1.7% (**Supplementary Table S2**).

5. Population genetic signals
We took note of signals from our population genetic analyses (particularly admixture graphs) that might plausibly be effects of contamination. This type of evidence, while potentially quite useful, should of course not be used in isolation. For our work, the primary example comes in the form of individuals with inferred excess allele-sharing with non-Africans. As described in more detail in **Supplementary Note 6**, the relevant individuals can be grouped well based on multiple criteria into those likely to have real admixture and those likely to have contamination. In the latter category are I2966 (Hora 1), for whom contamination was previously identified[61], and I13763 (Gishimangeda), who is discussed above. We note that for I13763, when we allow European-related admixture, the inferred proportion (9%) is quite similar to the implied contamination level from the sex chromosome ratio analysis. Overall, the fact that we observe close clustering and similar sources of ancestry for the ancient individuals across multiple population genetic analyses, and that their ancestry is highly divergent from almost all people living today (as potential contaminant sources), gives us more confidence that our results are not meaningfully affected by contamination.

Supplementary Note 6. Admixture graph fitting

Here, we provide additional details about our approach to building admixture graphs and the results we obtained from our admixture graph models.

Adapting the model from Lipson et al.[17] into our Model 1

We took as our starting point the admixture graph model proposed in Lipson et al.[17]. For the purposes of the present work, we simplified the set of populations by omitting several that were now less relevant: chimpanzee (as a deep outgroup), Shum Laka, Mende, and Lemande. This left Altai Neanderthal, ancient southern African foragers, the Mota individual, and present-day Mbuti, Aka, Agaw, Yoruba, and French. Again, for simplicity, we used the version of the model with an alternative formulation for the deep ancestry in the western African lineage (a single source rather than separate deep modern human and archaic ancestry). We used our new diploid data for Mota and increased sample sizes for Mbuti, Aka, Yoruba, and French by utilising data from Bergstrom et al.[114]. Finally, to create our Model 1, we added the highest-coverage representatives from the main genetic clusters of ancient eastern and south-central African foragers, namely I8808 (Jawuoyo, Kenya), I8821 (Kisese II, Tanzania), and I4426 (Fingira, Malawi). We modeled those three individuals as each having a mixture of ancestry related to Mota, to southern African foragers, and to central African foragers (specifically Mbuti), using the same three source nodes but in different proportions. (Both here and elsewhere, these admixture events were specified in the model as single pulses, but the inferences should be interpreted as total proportions of ancestry from each source carried by each individual and not necessarily as reflecting events happening at a single time). Although we did not know *a priori* whether this structure would provide a good fit to the data, we adopted it as our default parsimonious modeling assumption, with the expectation that we would revise it (particularly in larger models, with more constraint) as necessary based on the empirical results. In fact, aside from excess shared drift among spatially clustered individuals, we found that the three-shared-sources formulation provided a good fit both in Model 1 and for virtually all individuals in the expanded models. We also performed tests to confirm that all three components were necessary to provide a good fit to the data (see below).

As described in **Methods** and **Supplementary Note 7**, we used the new *qpfstats* program with the 'allsnps: YES' option to calculate the empirical *f*-statistics to use in fitting the admixture graph models. For Model 1, which contains all relatively high-coverage individuals and populations, we also fit a version without *qpfstats* and using only SNPs with no missing data, and the results were virtually identical (**Extended Data Figure 5**). The inferred topologies and parameters of our models are additionally very similar to those in Lipson et al.[17] for the populations in common. Admixture events that are not discussed in the sections below are Neanderthal ancestry to non-Africans (locked at 2% for simplicity), deep ancestry to the western African lineage (~20-25%), and western African-related ancestry to Aka (~60%).

Add-one procedure and building expanded models

When building the larger Models 2 and 3, we were guided by results obtained by adding additional forager individuals one-by-one first to Model 1 and then again to Model 2. Our procedure was to start with Model 1 (and later with Model 2) and then add each individual using a mixture of ancestry from the same three sources (in flexible proportions) as for the forager individuals already in the model. If the resulting model did not fit well, we noted which population relationships were responsible and used this information to guide our construction of Models 2 and 3 (results can be

found in **Supplementary Table S11**). The three main purposes of Model 2 were (1) to provide an intermediate admixture graph version with a size and minimum coverage level in between those of the relatively small Model 1 and the quite large Model 3; (2) to enable follow-up analyses with such an intermediate-scale model; and (3) to aid in construction of Model 3 via a second round of the add-one procedure. For these reasons, we generally aimed for a large geographic coverage in Model 2 while omitting lower-coverage individuals from multi-individual sites.

The majority of the model violation signals that we observe involve excess allele-sharing between individuals from the same or nearby sites. Of course, such a pattern is not surprising; our initial model setup with the same sources of ancestry for all of the ancient forager individuals does not account for the (probably expected) greater relatedness over shorter distances. The simplest way to build a model that explains these signals is to specify two (or more) individuals with excess allele-sharing as forming a clade descended from the same three-way admixture event involving Mota-, southern African-, and central African-related ancestry. (One alternative could be to separate the source nodes in the graph to allow genetic drift specific to one or more regions, e.g., specific to the Mota-related ancestry for the Kenya cluster). Such a form also proposes that the two individuals have the same proportions of ancestry from the three sources (to within statistical uncertainty). Empirically, we find that this is true in almost all instances (see below), so we feel it is a reasonable representation of our results. The full history of the three-way admixture and subsequent population structure was undoubtedly more complex, although our observations argue against prevalent recent long-range gene flow. For other signals that did not fall into this category, we believe they are evidence either of additional admixture events or contamination; we describe the relevant results in the following section.

Overall, we obtain highly concordant results between Models 1, 2, and 3. The inferred mixture proportions for the ancient forager individuals vary modestly among the models, likely indicating incomplete constraint, but the differences are still relatively minor (**Supplementary Table S9**). As expected from the greater number of populations being fit, the larger models have more significant residuals (i.e., deviations between predicted and observed *f*-statistics), with maxima of Z = 2.0, 3.0, and 3.7 for Models 1, 2, and 3. A small number of branches had inferred lengths of zero: shared drift along the western African-related lineage contributing ancestry to other populations (all three models), shared drift along the pastoralist-related lineage (Model 2), and terminal drift for French and Agaw (Model 3). These sections of the graph(s) are thus likely not to capture the exact histories of the relevant populations, but as none of them are central to our results, we chose not to explore additional complexity. Further details about the model results can be found in the sections that follow.

We imposed a coverage threshold of 0.05x (~60,000 SNPs covered) for the individuals included in Model 3, a level that we selected to give a high degree of comprehensiveness while omitting individuals with potentially lower data quality from our primary results. We also built an expanded admixture graph including the next five highest-coverage individuals (minimum 0.016x coverage, or ~19,000 SNPs). We could fit the ~5.2 ka Chencherere individual (I4422) with an independent three-way admixture event, the lower-coverage early Hora 1 individual (I19529) as a clade with I19528 (the lower-coverage early Hora 1 individual), and the three additional Kenya individuals within the existing Kenya clade (the two individuals from Nyarindi and the two from White Rock Point respectively most closely related to each other). All *f*-statistics agreed between predicted and

observed values to within Z = 4.5. While Z = 4.5 is nominally highly significant, the large number of populations in the model (and hence very large number of statistics being assessed) makes it plausible that the residuals are mostly if not all due to noise (although at present we lack a formal correction for multiple hypothesis testing).

As an additional investigation, we built an extended version of Model 2 (**Extended Data Figure 8**) in which we added present-day Hadza (one, with another omitted due to a very high proportion of recent admixture) and Sandawe (two, pooled together) individuals[8]. Both had evidence of complex ancestry (cf. following sections); our final model fit them as related to the ancient inland foragers from Tanzania, but with substantial admixture from food producers (ancestry related to both early pastoralists and farmers) as well as small proportions of additional ancestry related to the Mota individual (Hadza, ~9%) or to southern African foragers (Sandawe, ~4%). We hypothesise that the latter signals could in part reflect relatively recent events, but it is also possible that groups with similar ancestry proportions (before admixture from food producers) lived in either similar or different areas of eastern Africa in the past.

Detailed modeling of forager individuals

Broadly, our models describe the ancestry of the ancient eastern and south-central African foragers as a three-way gradient, with varying proportions derived from Mota-, southern African-, and central African-related sources. As noted in the main text, we performed tests to evaluate whether all three sources are necessary or whether some individuals could be more parsimoniously modeled using only two. Our strategy with these tests was to use representative individuals with high coverage and thus greater statistical power; given the inferred high degree of homogeneity in ancestry proportions within each sub-region, our null hypothesis would be that lower-coverage individuals would be similar in their proportions to closely related test individuals. Our primary focus was on the central African-related component, which is inferred to be the smallest of the three on average (especially so outside of the Lake Victoria region). For each test, we locked at zero the proportion of the component of interest (e.g., central African-related ancestry for the Kisese individual) in one of our admixture graph models and measured the significance of residual poorly fitted *f*-statistics reflecting that signal (e.g., un-modeled allele-sharing between Kisese and Mbuti). Using Model 1 as a starting point, we observed violations up to Z = 6.9, 5.0, and 7.7 when setting the central African-related ancestry to be zero for the Fingira, Kisese, and Jawuoyo individuals, respectively, and Z = 4.0 for southern African-related ancestry for Jawuoyo (we note though that the Fingira individual I4426 in Model 1 has excess central African-related ancestry relative to others from Malawi). With Model 2, we observed violations up to Z = 6.1 and 4.5 when setting the central African-related ancestry to be zero for the Tanzania clade or the Kalemba individual, and Z = 4.7 for southern African-related ancestry for the Kenya clade. For other individuals from Malawi, we performed tests using an alternative model formulation in which they are fit with shared rather than separate three-way admixture (see below), and we observed violations up to Z = 3.6, with a log-likelihood score > 30 units inferior. We confirmed as well that the score difference and most significant residuals are in fact slightly larger when using a model without sharing of Mbuti's older admixture event (see next section), despite the lower inferred proportion of central African-related ancestry (7.4% versus 9.0%).

We also note two other ways in which the results of these tests can be useful. One is that when performing tests with zero central African-related ancestry in one or more individuals, in addition

to model violations involving allele-sharing with Mbuti, we consistently observe signals involving allele-sharing with Aka (Z = 4.5, 3.3, and 4.5 for the three tests referenced above with Model 1, and Z = 3.7 for the Tanzania clade in Model 2). This is precisely the pattern we would expect for a component of central African-related ancestry carried by the eastern and south-central African foragers that is closer phylogenetically to Mbuti than to Aka. By contrast, if the gene flow had been in the other direction (i.e., eastern African-related ancestry carried by Mbuti), we would not expect to see the signals with Aka. Second, the Z-scores for residual statistics can be combined with the corresponding inferred ancestry proportions from the full models to approximate the level of uncertainty in the ancestry proportion estimates. For example, in Model 2, the Kisese individual is inferred to have ~12% central African-related ancestry, which, combined with a maximum Z = 6.1 residual when locked at zero, yields a standard error of ~2% (although we stress that this is only a rough procedure).

While we find that the model structure we have outlined thus far accounts for most of the variation among the forager individuals, signals of un-modeled allele-sharing for some of them led us to propose additional admixture events. First, two individuals (KPL001 from Kakapel in Kenya and I4426 from Fingira in Malawi) had excess allele-sharing with other ancient foragers buried at the same or nearby locations but also excess relatedness to Mbuti, which we believe reflects additional central African-related ancestry (inferred ~10-15% each). Given that this ancestry plausibly reflects more recent gene flow, we tested whether it would fit better if originating from a source phylogenetically closer to present-day Mbuti. For KPL001, it was (modestly) better, so we used that position in our final models, but for I4426, the fit was not improved, so we used the same source as for the primary central African-related ancestry in all of the forager individuals.

Next, several individuals had signals of excess allele-sharing with non-Africans, for which the two natural explanations would be either admixture from pastoralists or contamination in the data. We used several criteria when deciding which was more likely for each individual, including data quality metrics, dates of the burials, and inferred dates of admixture. For two individuals, we suspected contamination, namely I2966 (Hora 1) and I13763 (Gishimangeda). While the latter is undated, the former (~9 ka) well predates the documented arrival of pastoralism in the region. Both individuals also returned evidence of contamination via standard data authentication measures. For the purposes of the model, we added ‘dummy’ non-African-related admixture to fill the place of the contamination (inferred proportions of 6% and 9%, respectively), after which both individuals fit well.

For the four other individuals with the same signal, we propose that this is a reflection of true ancestry traced to gene flow from pastoralists with western Eurasian-related ancestry (Prendergast et al. 2019). Three of the four are relatively recent (max ~1.4 ka) individuals from coastal and island Tanzania and Kenya and do not have evidence of contamination. One (I0595 from Panga ya Saidi) was previously observed to be admixed in this way; we infer approximately 29% of his ancestry to be related to pastoralists and an additional 17% to be related to western Africans (likely due to gene flow from farmers). The individual from Makangale Cave (I1048) is inferred to have ~18% ancestry related to pastoralists, as well as a recent date of admixture (albeit with fairly high uncertainty: 79 +/- 24 generations in the past), while the individual from Kuumbi Cave (I0589) is inferred to have ~3% ancestry related to pastoralists. Finally, we also observe a similar signal for individual I4421 from Fingira (Malawi). The skeleton was hypothesised to be close in time to

I4422 from the same site (~5.2 ka, again likely too early for contact with pastoralists), but it is itself undated. However, in addition to the evidence of allele-sharing with non-Africans, we find that I4421 has no direct evidence of contamination and also a recent LD-based inferred date of admixture (10 +/- 2 generations in the past). Thus, we believe that this individual is more recent and does in fact carry a small proportion (~4%) of pastoralist-related ancestry. We model this ancestry in the admixture graphs as related to present-day Agaw, but with little or none of Agaw's Mota-related ancestry (except for I0595, for whom we used a source more closely related to Agaw based on signals of greater allele-sharing).

As noted above, we observe a strong concordance whereby individuals with excess allele-sharing (beyond the level predicted by their proportions of ancestry from the three primary source lineages) almost always have very similar ancestry proportions. The converse, however, is not always true, as in Malawi, most individuals have similar ancestry proportions but do not display excess allele-sharing. As a result, while the individuals from Kenya and Tanzania can be modeled naturally in three distinct clades (see above), the individuals from Malawi can be modeled approximately equally well either as a single clade (with extra admixture for two individuals) or as four separate clades (Chencherere, Fingira, early Hora 1, later Hora 1). Although we present the latter version as our preferred model, the former is in some ways more parsimonious, with fewer separate admixture parameters, and only a modestly worse fit (no more significant residuals and a log-likelihood score ~12 worse for an alternative version of Model 2; **Extended Data Figure 6**) despite the assumption of equal ancestry proportions for all individuals. However, even when we specify the Malawi individuals as belonging to a single clade, the inferred shared drift is negligible: exactly zero at the base of the clade (i.e., shared by all individuals), and only fractions of a unit along the internal branches. Thus, we feel that the structure is better captured by a model with separate admixture events for each of the four smaller clades. Moreover, this pattern provides evidence for our hypothesis of changes in mobility over time, with earlier episodes of gene flow plausibly leading to similar ancestry proportions across the region before increased isolation was responsible for the (relative) lack of excess relatedness among the sampled individuals. We also note that we model individual I10726 (from Kalemba in Zambia, ~150 km from Chencherere) with separate admixture events in both versions, given the lack of evidence for excess allele-sharing with any Malawi individuals combined with a lower proportion of Mota-related ancestry (e.g., f_4-statistics up to Z = 3.7 in **Supplementary Table S7**). Finally, we note a contrast with individual I1048 from Makangale Cave, who showed only weak evidence of excess allele-sharing with individual I0589 (Kuumbi Cave) in our add-one procedure (max Z = 2.2) but had both similar ancestry proportions to the other coastal/island individuals and greater inferred shared drift (2 units) when modeled together in a clade, which led us to present that version in our final model.

Relationships of the three primary forager ancestry sources to other sampled populations

On a broad scale, the admixture graph results provide information about the relationships between the ancient forager individuals in this study and surrounding groups (related to the Mota individual and to central and southern African foragers). First, with regard to the southern African-related ancestry carried by the eastern and south-central African foragers, we find that it split quite deeply along the lineage leading to the sampled ancient southern African foragers (i.e., the two are related but only distantly, with the source group not directly represented as yet in genetic studies). Although the methods used here do not allow conversions from units of genetic drift to calendar years (and the two are in general not directly proportional even along a single branch in the

admixture graph, unless the ancestral effective population size remained constant), we can gain some insight from relative depths of splits. In particular, we built a variant of Model 2 in which we included one present-day Ju|'hoansi individual, the majority of whose ancestry comes from a lineage that diverged from the ancient southern African foragers ~20-30 ka[11,115]. In our admixture graph, this split point is separated by ~10 drift units from the divergence of southern African foragers from other modern humans (probably at least 200 ka). By contrast, the source contributing to eastern and south-central African foragers is inferred to split only ~4 units from the deep divergence. We also note that if we constrain the Ju|'hoansi individual to have ancestry instead from the same southern African-related source as the eastern and south-central African foragers, the model is rejected (Z = 4.3; log-likelihood score ~100 worse than the preferred model), providing a positive control for our ability to detect ancestry from phylogenetically distinct lineages through the admixture graph procedure.

We also find that the Mota-related ancestry carried by the eastern and south-central African foragers was only deeply related to the Mota individual himself. In part, this conclusion is based on the same reasoning as above, namely that the split position comes only 3-4 drift units after the split of the Mota lineage from non-Africans. We also found that the best proxy for the Mota-related ancestry in the eastern and south-central Africans did not include any of the Mota-specific deep ancestry component. By contrast, the Mota-related ancestry carried by present-day Agaw (from Ethiopia) is inferred to be phylogenetically substantially closer to the Mota individual and also to share the deep component (residual max Z = 4.9 when not shared in a version of Model 1). Additionally, we tested whether some or all of the deep ancestry might actually be derived from the same southern African-related or central African-related sources that contributed to the other ancient individuals, and found that the best fit was indeed a different source that split around the same time as the deep southern and central lineages (~250-200 ka).

Finally, for the third, central African-related component, we infer its position to be closer to present-day Mbuti than to Aka, placing its split time at most ~50 ka. The fact that we observe modest excess central African-related ancestry in two individuals (with some evidence of a source phylogenetically even closer to Mbuti in the case of KPL001; see next section) possibly also suggests more recent interactions between groups from central and eastern/south-central Africa. The model from Lipson et al.[17] specified Mbuti as having admixture from two sources, one western African-related and one Agaw-related (but without a non-African-related component). With further study, we found that we were not able to assign the second source confidently to the Agaw lineage; its best-fitting position was found to be slightly along that lineage in Models 1 and 3 but slightly before the split from western Africans in Model 2. The depth of the split of that component then led us to consider the possibility that the admixture in Mbuti could have been relatively old, and perhaps shared with the central African-related source contributing to the eastern and south-central African foragers. And, indeed, while there were no individual residual statistics strongly rejecting either model, we found a better fit (log-likelihood score difference of ~18 for equivalent complexity) using an admixed central African-related source (~70% ancestry from the primary central African lineage). Otherwise, the models are very similar, although with somewhat lower inferred proportions of central African-related ancestry when the source is not specified as admixed (see next section). We also note that results from ALDER[116] in one-reference mode (using either Yoruba or Dinka) suggest at least ~15% recent admixture in Mbuti, and we infer ~11-13% western African ancestry in our admixture graph models.

One of the notable results that emerges from our analyses is that although we observe excess allele-sharing between some of the forager individuals, such signals are restricted to individuals buried in relatively close proximity, and similarly, we observe very few instances of excess allele sharing between the eastern and south-central African foragers and either the Mota individual or southern or central African foragers. In other words, our framework in which the same three sources are used to model the ancestry of the eastern and south-central African foragers provides a good approximation to the data. Two alternative possibilities (among others) could have been that (a) we might have observed greater allele sharing between the individuals from Kenya and the individuals from Tanzania relative to the individuals from Malawi and Zambia, or (b) we might have observed, for example, greater allele-sharing (even after accounting for different ancestry proportions) between southern African foragers and the individuals from Malawi and Zambia. The lack of such signals form part of the basis for our conclusion that the observed three-way ancestry gradient was a relatively old phenomenon and involved longer-range dispersals (related to the three primary sources of ancestry) on average than occurred by the Holocene. We also note that all three source lineages are best fit (in all three models) with positive shared drift branches, i.e., the southern African-related ancestry for each eastern and south-central African forager individual is more closely related to the ancestry carried by the other foragers than it is to the sampled ancient southern African forager individuals (and similarly for the other two sources, although we note that the constraint for the central African-related source is likely limited due to the smaller proportions).

Another specific observation that falls into the same category is the lack of excess relatedness (after accounting for ancestry proportions) among most of the individuals within Malawi and Zambia (see next section). Overall, our interpretation of our results is that recent interactions tended to be short range, and even more so in Malawi and Zambia than farther north. In theory, there could be less shared drift due to larger effective population sizes in some groups even if the scale of dispersal were similar. Empirically, however, we observe significant excess relatedness for individuals buried at both Hora 1 (closer together in time) and Fingira, showing that our methods do have power to detect such signals for the individuals in question. There could also potentially be some feature of our modeling approach that makes it difficult to detect low levels of excess allele-sharing, but in addition to the results discussed in the next section, we also infer multiple levels of structure within Kenya and Tanzania, with the greatest excess relatedness at the shortest distances (as in Malawi) but some amount of significant excess allele-sharing across the entire regions. Those results also show that our statistical power is sufficient with the available sequencing coverage to detect such signals.

Supplementary Note 7 *qpfstats*

Here we describe a recently released program, *qpfstats*, available as part of the ADMIXTOOLS software suite (https://github.com/DReichLab/AdmixTools). The program allows for estimation of a consistent set of *f*-statistics (statistics measuring allele-sharing between pairs, triples, or quadruples of populations; see Patterson et al.[117] for a full introduction) involving multiple individuals or populations with missing data, which is especially useful for applications such as admixture graph fitting that can involve many such populations simultaneously.

Background

The set of all *f*-statistics (f_2, f_3, and f_4) relating a collection of n populations forms a linear vector space of dimension $n(n-1)/2$. (The same is true of the set of expected values of the same statistics.) One basis, which is used in ADMIXTOOLS, is all statistics of form $f_3(O;A,B)$, where O is an outgroup and $A,B \neq O$ ($n-1$ of the statistics are in fact f_2-statistics, where $A = B$).
Any *f*-statistic f_i can be written as $f_i = \sum_j c_{ij} b_j$, where b_j are the basis statistics and c_{ij} are fixed constants determined by the linear dependence relationships. If the data are complete, with no missing genotypes, such equations will hold as identities. In the presence of missing data, we can evaluate all statistics on an intersection of the covered SNPs for all n populations, but this might be an impractically small set. Alternatively, we can compute each statistic separately on as many SNPs as possible, i.e., all SNPs with data for all of the populations that feature in it (as implemented via the option 'allsnps: YES' in ADMIXTOOLS). However, in this case, the equations above, while correct in expectation (assuming missing SNPs are an unbiased subset), will not be exact as computed from the data. How much of an issue this creates can differ from one analysis to another, but it appears to be especially problematic for admixture graph fitting[118].

One simple way in which non-overlapping SNPs have been used in the past has been to compute a single statistic $f_4(A, B; C, D)$ in which two of the populations (say C and D) have such limited coverage that even the number of SNPs available for both of those two is very low[119,120]. In this case, rather than estimate $f_4(A, B; C, D)$ directly, one can compute the difference $f_4(A, B; E, D) - f_4(A, B; E, C)$, which has the same expected value (as implemented in the *qp4diff* program). Here E is some other population with little missing data, allowing the two f_4-statistics to make use of many more (different) SNPs. The new technique described below can in some ways be thought of as an improved and generalised version of this idea.

New approach

Returning to the basis representation from above, we can write $f_i = \sum_j c_{ij}\beta_j + n_i$, where here we treat the empirical *f*-statistic f_i (computed on as many sites as possible) as a linear combination of variables β_j (for which we would like to solve) standing in for the basis elements, plus a noise term $n_i \sim N(0, \sigma_i^2)$. If there were no missing data, the system of such equations would be trivial, but computing different f_i on different sets of SNPs introduces noise, with a magnitude σ_i that we can estimate for each statistic by applying a block jackknife (at present we treat the n_i as independent, although in reality they will not be). We can then divide by σ_i to yield $\sum_j \frac{c_{ij}}{\sigma_i}\beta_j \approx f_i/\sigma_i$, and we solve the system for the β_j via least-squares, giving us our final estimates $\widehat{b_j} = \beta_j$ for the basis elements of the space of *f*-statistics. Moreover, we can compute these estimates within a second block jackknife to yield an error covariance matrix.

Finally, for applications (e.g., admixture graph fitting), we derive all needed *f*-statistics from the basis elements as $\sum_j c_{ij}\widehat{b_j}$. This ensures that the proper identities among *f*-statistics are obeyed, while simultaneously maximising coverage and smoothing out noise across multiple statistics.

We also introduce a correction to effectively ignore statistics for which a sample size correction cannot be accurately estimated, namely f_2- and f_3- statistics involving populations (for the latter, only as the target population) consisting of a single individual with pseudohaploid data. When the inbreeding correction is disabled (as it must be when using such populations), *qpfstats* flags populations with all homozygous genotypes; automatically sets standard errors of the relevant statistics to be very large (essentially infinite); solves the system as usual; and then, for any such statistics that are in the basis, automatically sets their estimated values to be zero and standard errors to be very large again in the final results. This process leads to what is technically an inconsistent set of statistics, but in practice the program is effectively ignoring those statistics that cannot be estimated from the data, with the standard error values meaning that the set is consistent to within the nominal statistical precision. When using the results for another application, the second program will use as much information as possible. In our work here, the primary observable effect is that terminal branch lengths in an admixture graph for single-individual pseudohaploid populations will come out as zero (as opposed to the very large – but also not meaningful – values reported without *qpfstats*).

Supplementary References

1. Mathieson, I. & Scally, A. What is ancestry? *PLOS Genet.* **16**, e1008624 (2020).
2. Prendergast, M. E. *et al.* Ancient DNA reveals a multistep spread of the first herders into sub-Saharan Africa. *Science* **365**, eaaw6275 (2019).
3. Schlebusch, C. Issues raised by use of ethnic-group names in genome study. *Nature* **464**, 487–487 (2010).
4. Henn, B. M., Steele, T. E. & Weaver, T. D. Clarifying distinct models of modern human origins in Africa. *Curr. Opin. Genet. Dev.* **53**, 148–156 (2018).
5. Chan, E. K. F. *et al.* Human origins in a southern African palaeo-wetland and first migrations. *Nature* **575**, 185–189 (2019).
6. Scheinfeldt, L. B. *et al.* Genomic evidence for shared common ancestry of East African hunting-gathering populations and insights into local adaptation. *Proc. Natl. Acad. Sci.* **116**, 4166–4175 (2019).
7. Gopalan, S. *et al.* Hunter-gatherer genomes reveal diverse demographic trajectories following the rise of farming in East Africa. *bioRxiv* 517730 (2019) doi:10.1101/517730.
8. Fan, S. *et al.* African evolutionary history inferred from whole genome sequence data of 44 indigenous African populations. *Genome Biol* **20**, 82 (2019).
9. Lachance, J. *et al.* Evolutionary history and adaptation from high-coverage whole-genome sequences of diverse African hunter-gatherers. *Cell* **150**, 457–469 (2012).
10. Lorente-Galdos, B. *et al.* Whole-genome sequence analysis of a Pan African set of samples reveals archaic gene flow from an extinct basal population of modern humans into sub-Saharan populations. *Genome Biol.* **20**, 77 (2019).
11. Pickrell, J. K. *et al.* The genetic prehistory of southern Africa. *Nat. Commun.* **3**, 1143 (2012).

12. Rito, T. *et al.* A dispersal of Homo sapiens from southern to eastern Africa immediately preceded the out-of-Africa migration. *Sci. Rep.* **9**, 4728 (2019).
13. Schlebusch, C. M. *et al.* Genomic variation in seven Khoe-San groups reveals adaptation and complex African history. *Science* **338**, 374–379 (2012).
14. Shriner, D., Tekola-Ayele, F., Adeyemo, A. & Rotimi, C. N. Genetic ancestry of Hadza and Sandawe peoples reveals ancient population structure in Africa. *Genome Biol. Evol.* **10**, 875–882 (2018).
15. Tishkoff, S. A. *et al.* The genetic structure and history of Africans and African Americans. *Science* **324**, 1035–1044 (2009).
16. Schlebusch, C. M. *et al.* Human origins in Southern African palaeo-wetlands? Strong claims from weak evidence. *J. Archaeol. Sci.* **130**, 105374 (2021).
17. Lipson, M. *et al.* Ancient West African foragers in the context of African population history. *Nature* **577**, 665–670 (2020).
18. Schlebusch, C. M. *et al.* Southern African ancient genomes estimate modern human divergence to 350,000 to 260,000 years ago. *Science* **358**, 652–655 (2017).
19. Skoglund, P. *et al.* Reconstructing prehistoric African population structure. *Cell* **171**, 59-71.e21 (2017).
20. McBrearty, S. & Brooks, A. S. The revolution that wasn't: a new interpretation of the origin of modern human behavior. *J. Hum. Evol.* **39**, 453–563 (2000).
21. Tryon, C. A. The Middle/Later Stone Age transition and cultural dynamics of late Pleistocene East Africa. *Evol. Anthropol. Issues News Rev.* **28**, 267–282 (2019).
22. Brooks, A. S. *et al.* Long-distance stone transport and pigment use in the earliest Middle Stone Age. *Science* **360**, 90 (2018).

23. Merrick, H. & Brown, F. H. Obsidian sources and patterns of source utilization in Kenya and northern Tanzania: some initial findings. *Afr. Archaeol. Rev.* **2**, 129–152 (1984).

24. Merrick, H., Brown, F. H. & Nash, W. P. Use and movement of obsidian in the Early and Middle Stone Ages of Kenya and northern Tanzania. in *Society, Culture and Technology in Africa* (ed. Childs, S. T.) 29–44 (MASCA, 1994).

25. Prendergast, M. E. The History of Eastern African Foragers. *Oxford Research Encyclopedia of African History* https://oxfordre.com/africanhistory/view/10.1093/acrefore/9780190277734.001.0001/acrefore-9780190277734-e-405 (2020) doi:10.1093/acrefore/9780190277734.013.405.

26. Ambrose, S. H. Small things remembered: Origins of early microlithic industries in sub-Saharan Africa. *Archaeol. Pap. Am. Anthropol. Assoc.* **12**, 9–29 (2002).

27. Stewart, B. A. *et al.* Ostrich eggshell bead strontium isotopes reveal persistent macroscale social networking across late Quaternary southern Africa. *Proc. Natl. Acad. Sci.* **117**, 6453–6462 (2020).

28. Tryon, C. A. & Faith, J. T. A demographic perspective on the Middle to Later Stone Age transition from Nasera rockshelter, Tanzania. *Philos. Trans. R. Soc. B Biol. Sci.* **371**, 20150238 (2016).

29. Archer, W. Carrying capacity, population density and the later Pleistocene expression of backed artefact manufacturing traditions in Africa. *Philos. Trans. R. Soc. B Biol. Sci.* **376**, 20190716 (2021).

30. Mackay, A., Stewart, B. A. & Chase, B. M. Coalescence and fragmentation in the late Pleistocene archaeology of southernmost Africa. *J. Hum. Evol.* **72**, 26–51 (2014).

31. Shipton, C. *et al.* The Middle to Later Stone Age transition at Panga ya Saidi, in the tropical coastal forest of eastern Africa. *J. Hum. Evol.* **153**, 102954 (2021).
32. Ambrose, S. H. Chronology of the Later Stone Age and food production in East Africa. *J. Archaeol. Sci.* **25**, 377–392 (1998).
33. French, J. C., Riris, P., Fernandéz-López de Pablo, J., Lozano, S. & Silva, F. A manifesto for palaeodemography in the twenty-first century. *Philos. Trans. R. Soc. B Biol. Sci.* **376**, 20190707 (2021).
34. Timmermann, A. & Friedrich, T. Late Pleistocene climate drivers of early human migration. *Nature* **538**, 92–95 (2016).
35. Cox, M. P. *et al.* Autosomal resequence data reveal Late Stone Age signals of population expansion in sub-Saharan African foraging and farming populations. *PloS One* **4**, e6366 (2009).
36. Hitchcock, R. K. Foragers and food production in Africa: A cross-cultural and analytical perspective. *World J. Agric. Soil Sci.* **1**, (2019).
37. Hitchcock, R. K., Sapignoli, M. & Babchuk, W. A. Settler colonialism, conflicts, and genocide: interactions between hunter-gatherers and settlers in Kenya, and Zimbabwe and northern Botswana. *Settl. Colon. Stud.* **5**, 40–65 (2015).
38. Hill, K. R. *et al.* Co-residence patterns in hunter-gatherer societies show unique human social structure. *Science* **331**, 1286–1289 (2011).
39. Lane, P. J. Hunter-gatherer-fishers, ethnoarchaeology, and analogical Reasoning. in *The Oxford Handbook of the Archaeology and Anthropology of Hunter-Gatherers* (eds. Cummings, V., Jordan, P. & Zvelebil, M.) 104–150 (Oxford University Press, 2014).

40. Scerri, E. M. L. *et al.* Did our species evolve in subdivided populations across Africa, and why does it matter? *Trends Ecol. Evol.* **33**, 582–594 (2018).

41. Harvati, K. *et al.* The Later Stone Age calvaria from Iwo Eleru, Nigeria: morphology and chronology. *PLOS ONE* **6**, e24024 (2011).

42. Mirazón Lahr, M. The shaping of human diversity: filters, boundaries and transitions. *Philos. Trans. R. Soc. B Biol. Sci.* **371**, 20150241 (2016).

43. Crevecoeur, I., Brooks, A., Ribot, I., Cornelissen, E. & Semal, P. Late Stone Age human remains from Ishango (Democratic Republic of Congo): New insights on Late Pleistocene modern human diversity in Africa. *J. Hum. Evol.* **96**, 35–57 (2016).

44. Morris, A. G. & Ribot, I. Morphometric cranial identity of prehistoric Malawians in the light of sub-Saharan African diversity. *Am. J. Phys. Anthropol.* **130**, 10–25 (2006).

45. Morris, A. G. Isolation and the origin of the khoisan: Late pleistocene and early holocene human evolution at the southern end of Africa. *Hum. Evol.* **17**, 231–240 (2002).

46. Tryon, C. A. & Ranhorn, K. L. Raw material and regionalization in Stone Age eastern Africa. in *Culture History and Convergent Evolution* 143–156 (Springer, 2020).

47. Barham, L. & Mitchell, P. *The First Africans: African archaeology from the earliest toolmarkers to most recent foragers*. (Cambridge University Press, 2008).

48. Thompson, J. C. Faunal analysis in African archaeology. *Oxford Research Encyclopedia of Anthropology* https://oxfordre.com/anthropology/view/10.1093/acrefore/9780190854584.001.0001/acrefore-9780190854584-e-44 (2020) doi:10.1093/acrefore/9780190854584.013.44.

49. Güldemann, T. & Elderkin, E. D. On the external genealogical relationships of the Khoe family. in *Khoisan Language and Linguistics: the Riezlern Symposium 2003* vol. 17 15–52 (Rüdiger Köppe, 2010).

50. Güldemann, T. "Khoisan" linguistic classification today. in *Beyond 'Khoisan': historical relations in the Kalahari Basin* 1–41 (John Benjamins, 2014).

51. Dyson-Hudson, R. & Smith, E. A. Human territoriality: an ecological reassessment. *Am. Anthropol.* **80**, 21–41 (1978).

52. Wilmsen, E. N. Interaction, spacing behavior, and the organization of hunting bands. *J. Anthropol. Res.* **29**, 1–31 (1973).

53. Kelly, R. L. *The Lifeways of Hunter-Gatherers: The Foraging Spectrum*. (Cambridge University Press, 2013).

54. Prendergast, M. E. & Sawchuk, E. Boots on the ground in Africa's ancient DNA 'revolution': archaeological perspectives on ethics and best practices. *Antiquity* **92**, 803–815 (2018).

55. Gallego Llorente, M. *et al.* Ancient Ethiopian genome reveals extensive Eurasian admixture in Eastern Africa. *Science* **350**, 820 (2015).

56. Arthur, J. W. *et al.* The Transition from hunting–gathering to food production in the Gamo Highlands of Southern Ethiopia. *Afr. Archaeol. Rev.* **36**, 5–65 (2019).

57. Bronk Ramsey, C. Bayesian analysis of radiocarbon dates. *Radiocarbon* **51**, 337–360 (2009).

58. Marsh, E. J. *et al.* IntCal, SHCal, or a mixed Curve? Choosing a ^{14}C calibration curve for archaeological and paleoenvironmental records from tropical South America. *Radiocarbon* **60**, 925–940 (2018).

59. Reimer, P. J. *et al.* The IntCal20 northern hemisphere radiocarbon age calibration curve (0–55 cal kBP). *Radiocarbon* **62**, 725–757 (2020).

60. Hogg, A. G. *et al.* SHCal20 southern hemisphere calibration, 0–55,000 Years cal BP. *Radiocarbon* **62**, 759–778 (2020).

61. Skoglund, P. *et al.* Reconstructing prehistoric African population structure. *Cell* **171**, 59-71.e21 (2017).

62. Sandelowsky, B. Later Stone Age lithic assemblages from Malawi and their technologies. (University of California, Berkeley, 1972).

63. Clark, J. D. Prehistory in Nyasaland. *Nyasal. J.* **9**, 92–119 (1956).

64. Morris, A. G. & Ribot, I. Morphometric cranial identity of prehistoric Malawians in the light of sub-Saharan African diversity. *Am. J. Phys. Anthropol.* **130**, 10–25 (2006).

65. Goldberg, P. & Macphail, R. I. Short contribution: Strategies and techniques in collecting micromorphology samples. *Geoarchaeology* **18**, 571–578 (2003).

66. Courty, M. A., Goldberg, P. & Macphail, R. *Soils and micromorphology in Archaeology, Cambridge University Press, 1989 (R. Nisbet)*. (Cambridge University Press, 1989).

67. Stoops, G. *Guidelines for Analysis and Description of Soil and Regolith Thin Sections*. (Soil Science Society of America, 2003).

68. Villagran, X. S., Strauss, A., Miller, C., Ligouis, B. & Oliveira, R. Buried in ashes: Site formation processes at Lapa do Santo rockshelter, east-central Brazil. *J. Archaeol. Sci.* **77**, 10–34 (2017).

69. Phillipson, D. W. Zambian rock paintings. *World Archaeol.* **3**, 313–327 (1972).

70. Phillipson, D. W. The prehistoric succession in Eastern Zambia: a preliminary report. *Azania Archaeol. Res. Afr.* **8**, 3–24 (1973).

71. Phillipson, D. *The Prehistory of Eastern Zambia*. (British Institute in Eastern Africa, 1976).
72. Robinson, J. R. Thinking locally: Environmental reconstruction of Middle and Later Stone Age archaeological sites in Ethiopia, Kenya, and Zambia based on ungulate stable isotopes. *J. Hum. Evol.* **106**, 19–37 (2017).
73. Miller, S. F. The age of Nachikufan industries in Zambia. *South Afr. Archaeol. Bull.* **26**, 143–146 (1971).
74. Clark, J. D. The newly discovered Nachikufu Culture of Northern Rhodesia and the possible origin of certain elements of the South African Smithfield Culture: Presidential Address. *South Afr. Archaeol. Bull.* **5**, 86–98 (1950).
75. Kusimba, S. B. *Hunter-gatherer-fishers of eastern and south-central Africa since 20,000 years ago*. (Oxford University Press, 2013). doi:10.1093/oxfordhb/9780199569885.013.0032.
76. de Villiers, H. Human skeletal material. in *The Prehistory of Eastern Zambia* (ed. Phillipson, D. W.) 163–165 (British Institute in Eastern Africa, 1976).
77. de Villiers, H. & Fatti, L. The antiquity of the negro. 19.
78. Masao, F. T. *The rock art of Kondoa and Singida: a comparative description*. (National Museums of Tanzania, 1982).
79. Bwasiri, E. J. & Smith, B. W. The rock art of Kondoa District, Tanzania. *Azania Archaeol. Res. Afr.* **50**, 437–459 (2015).
80. Campbell, A. & Coulson, D. Kondoa: World heritage rock painting site. *Adoranten* 5 (2012).
81. Leakey, M. D. *Africa's vanishing art: the rock paintings of Tanzania*. (Doubleday Books, 1983).

82. Leakey, L. S. B. *Stone Age Africa: an outline of prehistory in Africa*. (Oxford University Press, H. Milford, 1936).

83. Leakey, L. S. The archaeological aspect of the Tanganyika paintings. *Tanganyika Notes Rec.* **29**, 15 (1950).

84. Leakey, L. S. B. Preliminary notes on a survey of prehistoric art in Tanganyika. *Actes Congrès Panafricain Préhistoire IIe Sess. Alger* 723–724 (1955).

85. Inskeep, R. The age of the Kondoa Rock paintings in light of recent excavations at Kisese II rock shelter. in *Actes du IVe Congrès Panafricain de Préhistoire et de l'ectude du Quaternaire* (eds. Mortelmans, G. & Nenquin, J.) vol. 40 259–256 (Annales de Musée Royal de L'Afrique Centrale, 1962).

86. Laird, M. F. *et al.* Human burials at the Kisese II rockshelter, Tanzania. *Am. J. Phys. Anthropol.* **175**, 187–200 (2021).

87. Niespolo, E. M. *et al.* Carbon, nitrogen, and oxygen isotopes of ostrich eggshells provide site-scale Pleistocene-Holocene paleoenvironmental records for eastern African archaeological sites. *Quat. Sci. Rev.* **230**, 106142 (2020).

88. Ranhorn, K. *et al.* Kisese II rockshelter in Kondoa (Tanzania). in *Handbook of Pleistocene Archaeology of Africa: Hominin Behavior, Geography, and Chronology* (eds. Beyin, A., Wright, D., Wilkins, J., Bouzouggar, A. & Olszewski, D.) (in press).

89. Ranhorn, K. *et al.* Late Pleistocene-Holocene archaeology and paleoenvironments of the Kondoa region, Tanzania. *Bull. Société Préhistorique Fr.* (in review).

90. Tryon, C. A. *et al.* Middle and Later Stone Age chronology of Kisese II rockshelter (UNESCO World Heritage Kondoa Rock-Art Sites), Tanzania. *PLoS ONE* **13**, e0192029-24 (2018).

91. Tryon, C. A., Lewis, J. E. & Ranhorn, K. Excavating the archives: The 1956 excavation of the Late Pleistocene-Holocene sequence at Kisese II (Tanzania). in *Modern Human Origins and Dispersal* (eds. Sahle, Y., Reyes-Centeno, H. & Bentz, C.) 233–257 (Kerns Verlag, 2019).
92. Schmidt, P. R. & Pikirayi, I. *Community archaeology and heritage in Africa: Decolonizing practice*. (Routledge, 2016).
93. Stuiver, M., Reimer, P. & Reimer, R. *CALIB 8.2 Radiocarbon Calibration.* (2021).
94. Callow, W. J., Baker, M. J. & Pritchard, D. H. National Physical Laboratory Radiocarbon Measurements II. *Radiocarbon* **6**, 25–30 (1964).
95. Willoughby, P. R., Biittner, K. M., Bushozi, P. M. & Miller, J. M. A German rifle casing and Chief Mkwawa of the Wahehe: the colonial and post-colonial significance of Mlambalasi Rockshelter, Iringa Region, Tanzania. *J. Afr. Archaeol.* **1**, 1–13 (2019).
96. Biittner, K. M. *et al.* Excavations at Mlambalasi Rockshelter: A Terminal Pleistocene to Recent Iron Age record in Southern Tanzania. *Afr. Archaeol. Rev.* **34**, 275–295 (2017).
97. Sawchuk, E. A. & Willoughby, P. R. Terminal Pleistocene Later Stone Age human remains from the Mlambalasi Rock Shelter, Iringa Region, Southern Tanzania. *Int. J. Osteoarchaeol.* **25**, 593–607 (2015).
98. McClure, S. B., Puchol, O. G. & Culleton, B. J. AMS dating of human bone from Cova de la Pastora: new evidence of ritual continuity in the prehistory of eastern Spain. *Radiocarbon* **52**, 25–32 (2010).
99. Lohse, J., Culleton, B., Black, S. & Kennett, D. A precise chronology of middle to late Holocene bison exploitation in the far southern Great Plains. *Index Tex. Archaeol. Open Access Grey Lit. Lone Star State* (2014) doi:10.21112/ita.2014.1.78.

100. van Klinken, G. J. Bone collagen quality indicators for palaeodietary and radiocarbon measurements. *J. Archaeol. Sci.* **26**, 687–695 (1999).

101. Ambrose, S. H. Preparation and characterization of bone and tooth collagen for isotopic analysis. *J. Archaeol. Sci.* **17**, 431–451 (1990).

102. Santos, G. M., Southon, J. R., Druffel-Rodriguez, K. C., Griffin, S. & Mazon, M. Magnesium perchlorate as an alternative water trap in AMS graphite sample preparation: A report on sample preparation at Kccams at the University of California, Irvine. *Radiocarbon* **46**, 165–173 (2004).

103. Stuiver, M. & Polach, H. A. Discussion reporting of 14C dates. *Radiocarbon* **19**, 355–363 (1977).

104. Jones, M. B., Brandt, S. A., Henry, E. R. & Ambrose, S. H. Improved ostrich eggshell and ungulate tooth enamel radiocarbon dating methods reveal Later Stone Age occupation in arid MIS 2 southern Somalia. *J. Archaeol. Sci. Rep.* **36**, 102844 (2021).

105. Krueger, H. W. Exchange of carbon with biological apatite. *J. Archaeol. Sci.* **18**, 355–361 (1991).

106. Balter, V., Saliège, J.-F., Bocherens, H. & Person, A. Evidence of physico–chemical and isotopic modifications in archaeological bones during controlled acid etching. *Archaeometry* **44**, 329–336 (2002).

107. Cherkinsky, A. Can we get a good radiocarbon age from “bad bone”? Determining the reliability of radiocarbon age from bioapatite. *Radiocarbon* **51**, 647–655 (2009).

108. Zazzo, A. Bone and enamel carbonate diagenesis: A radiocarbon prospective. *Palaeogeogr. Palaeoclimatol. Palaeoecol.* **416**, 168–178 (2014).

109. Balasse, M., Ambrose, S. H., Smith, A. B. & Price, T. D. The seasonal mobility model for prehistoric herders in the south-western Cape of South Africa assessed by isotopic analysis of sheep tooth enamel. *J. Archaeol. Sci.* **29**, 917–932 (2002).

110. Rohland, N., Harney, E., Mallick, S., Nordenfelt, S. & Reich, D. Partial uracil–DNA–glycosylase treatment for screening of ancient DNA. *Philos. Trans. R. Soc. B Biol. Sci.* **370**, 20130624 (2015).

111. Sawyer, S., Krause, J., Guschanski, K., Savolainen, V. & Pääbo, S. Temporal Patterns of Nucleotide Misincorporations and DNA Fragmentation in Ancient DNA. *PLOS ONE* **7**, e34131 (2012).

112. Meyer, M. *et al.* A High-Coverage Genome Sequence from an Archaic Denisovan Individual. *Science* **338**, 222–226 (2012).

113. Gansauge, M.-T. & Meyer, M. Single-stranded DNA library preparation for the sequencing of ancient or damaged DNA. *Nat. Protoc.* **8**, 737–748 (2013).

114. Bergström, A. *et al.* Insights into human genetic variation and population history from 929 diverse genomes. *Science* **367**, eaay5012 (2020).

115. Mallick, S. *et al.* The Simons Genome Diversity Project: 300 genomes from 142 diverse populations. *Nature* **538**, 201–206 (2016).

116. Loh, P.-R. *et al.* Inferring admixture histories of human populations using linkage disequilibrium. *Genetics* **193**, 1233–1254 (2013).

117. Patterson, N. *et al.* Ancient admixture in human history. *Genetics* **192**, 1065–1093 (2012).

118. Lipson, M. Applying f4-statistics and admixture graphs: Theory and examples. *Mol. Ecol. Resour.* **20**, 1658–1667 (2020).

119. Lipson, M. *et al.* Population turnover in remote Oceania shortly after initial settlement. *Curr. Biol.* **28**, 1157-1165.e7 (2018).

120. Shinde, V. *et al.* An ancient Harappan genome lacks ancestry from steppe pastoralists or Iranian farmers. *Cell* **179**, 729-735.e10 (2019).
