# Supplementary material for: Ancient DNA and deep population structure in sub-Saharan African foragers
Source: Nature. 2022 Feb 23;603(7900):290–6. doi: 10.1038/s41586-022-04430-9 (PMC8907066; doi:10.1038/s41586-022-04430-9)
Supplement: Supplementary file 2 — Reporting Summary [file 41586_2022_4430_MOESM2_ESM.pdf]

nature portfolio

Corresponding author(s): Mary E. Prendergast

Last updated by author(s): December 13, 2021

Reporting Summary

Nature Portfolio wishes to improve the reproducibility of the work that we publish. This form provides structure for consistency and transparency in reporting. For further information on Nature Portfolio policies, see our Editorial Policies and the Editorial Policy Checklist.

Statistics

For all statistical analyses, confirm that the following items are present in the figure legend, table legend, main text, or Methods section.

n/a	Confirmed	
☐	☒	The exact sample size (*n*) for each experimental group/condition, given as a discrete number and unit of measurement
☐	☒	A statement on whether measurements were taken from distinct samples or whether the same sample was measured repeatedly
☐	☒	The statistical test(s) used AND whether they are one- or two-sided<br>*Only common tests should be described solely by name; describe more complex techniques in the Methods section.*
☒	☐	A description of all covariates tested
☐	☒	A description of any assumptions or corrections, such as tests of normality and adjustment for multiple comparisons
☐	☒	A full description of the statistical parameters including central tendency (e.g. means) or other basic estimates (e.g. regression coefficient) AND variation (e.g. standard deviation) or associated estimates of uncertainty (e.g. confidence intervals)
☐	☒	For null hypothesis testing, the test statistic (e.g. *F*, *t*, *r*) with confidence intervals, effect sizes, degrees of freedom and *P* value noted<br>*Give P values as exact values whenever suitable.*
☒	☐	For Bayesian analysis, information on the choice of priors and Markov chain Monte Carlo settings
☒	☐	For hierarchical and complex designs, identification of the appropriate level for tests and full reporting of outcomes
☒	☐	Estimates of effect sizes (e.g. Cohen's *d*, Pearson's *r*), indicating how they were calculated

Our web collection on statistics for biologists contains articles on many of the points above.

Software and code

Policy information about availability of computer code

Data collection	BWA v0.6.1, HaploGrep2 v2.1.19, cTools v11, contamMix v1.0-10, ANGSD v0.923, other bioinformatics tools and data workflows (https://github.com/DReichLab/ADNA-Tools and https://github.com/DReichLab/adna-workflow), OxCal v4.4
Data analysis	ADMIXTOOLS v7.0.2 (includes new qpfstats software), EIGENSOFT v7.2.1, hapROH v0.3, DATES v1

For manuscripts utilizing custom algorithms or software that are central to the research but not yet described in published literature, software must be made available to editors and reviewers. We strongly encourage code deposition in a community repository (e.g. GitHub). See the Nature Portfolio guidelines for submitting code & software for further information.

Data

Policy information about availability of data

All manuscripts must include a data availability statement. This statement should provide the following information, where applicable:

- Accession codes, unique identifiers, or web links for publicly available datasets
- A description of any restrictions on data availability
- For clinical datasets or third party data, please ensure that the statement adheres to our policy

The aligned sequences are available through the European Nucleotide Archive under accession number PRJEB49291. Genotype data used in analysis are available at https://reich.hms.harvard.edu/datasets. Any other relevant data are available from the corresponding authors upon reasonable request.

Field-specific reporting

Please select the one below that is the best fit for your research. If you are not sure, read the appropriate sections before making your selection.

☐ Life sciences ☐ Behavioural & social sciences ☒ Ecological, evolutionary & environmental sciences

For a reference copy of the document with all sections, see nature.com/documents/nr-reporting-summary-flat.pdf

Ecological, evolutionary & environmental sciences study design

All studies must disclose on these points even when the disclosure is negative.

Study description	Population genetic analyses were performed on DNA data generated from ancient human skeletons as well as present-day individuals. Historical relationships were inferred primarily from allele-sharing patterns across populations, computed using genome-wide SNP genotypes.
Research sample	Six newly reported ancient human individuals buried in what are now Zambia, Malawi, and Tanzania; 28 previously published ancient individuals (15 with increased sequencing coverage in this study); published comparative data from present-day groups. Ancient DNA data are severely lacking from forager groups from sub-Saharan Africa, so we aimed to sample all the ancient skeletal remains associated with foraging in Later Stone Age archaeological contexts that we could access while respecting ancient DNA ethics guidelines that have the goal of preserving remains for future analysis, with the goal of filling in gaps in knowledge. We recognize that we were not able to sample from the great majority of contexts and there remain many gaps in available ancient DNA data from Africa.
Sampling strategy	We tested a total of 31 skeletal samples and obtained working data from six. We targeted approximately 1.2 million genome-wide SNPs in generating DNA data, which effectively cover almost all independent loci in the genome (due to linkage disequilibrium) and provide good power in population history analyses. We generated whole genome sequencing data for individuals for which the proportion of human DNA was high enough to make this possible, namely the individual from Mota Cave, Ethiopia.
Data collection	DNA from the ancient remains was extracted, sequenced, and processed into SNP genotype calls in the laboratory of D.R.
Timing and spatial scale	Ancient individuals were sampled from archaeological sites in eastern and south-central Africa with evidence for foraging lifeways and in association with Later Stone Age material culture. We attempted to sample as comprehensively as possible within this framework but due to the limitation in sample availability there remain substantial gaps in space and time.
Data exclusions	As noted above, the majority of the skeletal samples did not yield working data as assessed by standard ancient DNA quality criteria. In our primary admixture graph-based analyses, we restricted to individuals with sufficient sequencing coverage (> 0.05x) to give meaningful results.
Reproducibility	The genetic findings were reproduced for many samples through making multiple ancient DNA libraries which yielded consistent findings about genetic relatedness patterns.
Randomization	Most analyses of ancient individuals were done one-by-one, but in some cases subgroups were defined based on location and/or time period, or (for present-day individuals) ethno-linguistic groups.
Blinding	Blinding was not possible for this study due to the importance of co-analyzing each genetic datapoint closely in concert with the archaeological data. We performed all analyses, however, based on testing the null assumption (disproved in all case) of symmetrical genetic relationship of each individual to all other individuals.

Did the study involve field work? ☐ Yes ☒ No

Reporting for specific materials, systems and methods

We require information from authors about some types of materials, experimental systems and methods used in many studies. Here, indicate whether each material, system or method listed is relevant to your study. If you are not sure if a list item applies to your research, read the appropriate section before selecting a response.

Materials & experimental systems

n/a	Involved in the study	
☒	☐	Antibodies
☒	☐	Eukaryotic cell lines
☐	☒	Palaeontology and archaeology
☒	☐	Animals and other organisms
☒	☐	Human research participants
☒	☐	Clinical data
☒	☐	Dual use research of concern

Methods

n/a	Involved in the study	
☒	☐	ChIP-seq
☒	☐	Flow cytometry
☒	☐	MRI-based neuroimaging

Palaeontology and Archaeology

Specimen provenance

Malawi:
The two Hora 1 burials were recovered in 2019 and are curated by the Malawi Department of Museums and Monuments (formerly Department of Antiquities), now under the Ministry of Youth, Sports, and Culture. Additional sampled individuals from Mazinga and Hora were recovered in 2017 and 2018. The individuals from Fingira and Mtuzi/Chencherere II were recovered during 2016 fieldwork or retrieved from the National Repository in Nguludi, respectively. Permission for the research, including both excavation and ancient DNA protocols for both failed and successful samples, was provided under permits A/III/3.3/70, A/III/3.3/71, AD/23/56, and NCST/RTT/2/6. Export was provided under A/1/1/1/3.6/50, A/1/1/1/3.6/44, A/II/1.5/33, and MHQ/CUL/1/04/2.

Zambia:
Skeletal remains from Kalemba Rockshelter are curated at Livingstone Museum. Permissions to conduct research and to export skeletal remains for destructive sampling were received from the Director of the Livingstone Museum and from the National Heritage Conservation Commission (Permit NHCC/8WR/004/17).

Tanzania/Kenya:
For Kisese II Rockshelter, the site is located in Tanzania, but burials are currently curated in National Museums of Kenya in Nairobi. Permissions for aDNA sampling obtained in Kenya from the National Commission for Science, Technology, and Innovation (NACOSTI permit P/17/34239/17088), and through affiliation with the National Museums of Kenya (NMK); and in Tanzania from the Commission for Science and Technology (COSTECH permits 2017-220/221/222-NA-2012-50). Permission to export the skeletal samples from Kenya for destructive sampling was issued by the Cabinet Secretary, Ministry of Sports and Heritage, Kenya.

For Mlambalasi Rockshelter, the sampled burials are curated at the National Museum and House of Culture in Dar es Salaam, Tanzania. Permission to sample these remains was granted by the Commission for Science and Technology (COSTECH permits 2017-220/221/222-NA-2012-50), through affiliation with the National Museums of Tanzania (NMT). Permission to export the skeletal samples for destructive sampling was granted by the Division of Antiquities, Ministry of Natural Resources and Tourism (Export License 03/2018/2019).

Specimen deposition

Skeletal tissue samples exported from Kenya were repatriated to the National Museum of Kenya (NMK) in Nairobi in October 2017 and June 2019. While no intact tissue samples remain outside the country, remaining powder, DNA extracts, and libraries remain under curation at the Reich Laboratory at Harvard University as agreed upon with the NMK Head of Earth Sciences. All skeletal tissue samples exported from Tanzania were repatriated to the NMT in May 2019, in keeping with a Memorandum of Agreement (MOA), which allows for curation of remaining powder, DNA extracts, and libraries in the Reich Laboratory. All skeletal tissue samples from the Livingstone Museum were repatriated to that institution in June 2018, except for tissue remaining after radiocarbon dating, which was repatriated in June 2019. No intact tissue samples remain outside of Malawi, and remaining powder, DNA extracts, and libraries remain under curation at the Reich Laboratory at Harvard University.

Dating methods

Three new dates are provided, one on dental enamel and two on bone collagen. All three dates were obtained at the Pennsylvania State University (PSU) Radiocarbon Laboratory, via accelerator mass spectrometry (AMS). Details of sample pretreatment and radiocarbon dating procedures are provided in Supplementary Note 3. Radiocarbon ages were calibrated using OxCal version 4.4, employing a uniform prior (U(0,100)) that allows the program to model an unspecified mixture of two curves: IntCal20 and SHCal20.

☒ Tick this box to confirm that the raw and calibrated dates are available in the paper or in Supplementary Information.

Ethics oversight

Detailed proposals including protocols for minimally destructive sampling were provided to and approved by the permit-granting and curating authorities listed above: In Malawi, the Malawi Department of Museums and Monuments (formerly Department of Antiquities); in Zambia, the Livingstone Museum and the National Heritage Conservation Commission; in Tanzania, the Commission on Science and Technology and National Museum of Tanzania; and in Kenya, the National Commission for Science, Technology, and Innovation, and the National Museums of Kenya.

Note that full information on the approval of the study protocol must also be provided in the manuscript.
